# Twin-AI: Intelligent Barrier Eddy Current Separator with Digital Twin and AI Integration

**DOI:** 10.3390/s25154731

**Published:** 2025-07-31

**Authors:** Shohreh Kia, Johannes B. Mayer, Erik Westphal, Benjamin Leiding

**Affiliations:** 1 Institute for Software and Systems Engineering, Clausthal University of Technology, 38678 Clausthal-Zellerfeld, Germany; johannes.mayer@tu-clausthal.de; 2 ESD Elektro Systemtechnik Dargun GmbH, 17159 Dargun, Germany; erik.westphal@esd-dargun.de

**Keywords:** barrier eddy current separator (BECS), digital twin, Industry 4.0, Internet of Things (IoT)

## Abstract

The current paper presents a comprehensive intelligent system designed to optimize the performance of a barrier eddy current separator (BECS), comprising a conveyor belt, a vibration feeder, and a magnetic drum. This system was trained and validated on real-world industrial data gathered directly from the working separator under 81 different operational scenarios. The intelligent models were used to recommend optimal settings for drum speed, belt speed, vibration intensity, and drum angle, thereby maximizing separation quality and minimizing energy consumption. the smart separation module utilizes YOLOv11n-seg and achieves a mean average precision (mAP) of 0.838 across 7163 industrial instances from aluminum, copper, and plastic materials. For shape classification (sharp vs. smooth), the model reached 91.8% accuracy across 1105 annotated samples. Furthermore, the thermal monitoring unit can detect iron contamination by analyzing temperature anomalies. Scenarios with iron showed a maximum temperature increase of over 20 °C compared to clean materials, with a detection response time of under 2.5 s. The architecture integrates a Digital Twin using Azure Digital Twins to virtually mirror the system, enabling real-time tracking, behavior simulation, and remote updates. A full connection with the PLC has been implemented, allowing the AI-driven system to adjust physical parameters autonomously. This combination of AI, IoT, and digital twin technologies delivers a reliable and scalable solution for enhanced separation quality, improved operational safety, and predictive maintenance in industrial recycling environments.

## 1. Introduction

The increasing global consumption of electronic devices, coupled with their short life cycles, has led to a massive surge in electronic waste (e-waste), making it the fastest-growing waste stream worldwide [1]. In 2019 alone, over 53.6 million metric tons of e-waste were generated globally, and this number is projected to exceed 74 million tons by 2030 [1]. E-waste contains valuable resources such as aluminum, copper, gold, silver, palladium, and rare earth elements—many of which are critical raw materials for modern industries, including renewable energy, aerospace, automotive, telecommunications, and semiconductor manufacturing [2,3].

The effective recycling of these metals plays a crucial role in reducing dependency on virgin resources, lowering environmental pollution, and supporting the circular economy. However, the recycling of e-waste poses considerable challenges. Traditional recycling methods often rely on manual labor, inefficient shredding processes, and basic magnetic separation techniques, which may not effectively separate non-ferrous or coated materials [4]. Additionally, the improper handling of e-waste can release hazardous substances such as lead, mercury, and cadmium, posing serious environmental and health risks [5]. Therefore, the development of intelligent, efficient, and safe sorting systems is crucial to meet the growing demands for recycling.

Among the most promising technologies for non-ferrous metal separation is the barrier eddy current separator (BECS). These systems are extensively used in industrial recycling plants to separate conductive, non-magnetic metals—such as aluminum, copper, and brass—from complex waste mixtures [6,7,8]. The BECS operates based on the principle of eddy current repulsion, where a high-speed rotating magnetic drum induces eddy currents in conductive particles, generating opposing magnetic fields that deflect the particles into separate bins.

**Figure 1:**
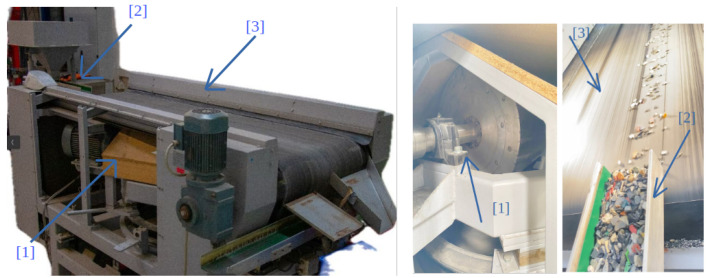
Barrier eddy current separator: (1) magnetic drum; (2) vibration feeder; and (3) conveyor belt.

As shown in Figure 1, a typical BECS comprises three main components: (1) a magnetic drum, (2) a vibration feeder, and (3) a conveyor belt. The vibration feeder ensures the uniform distribution of materials, the conveyor transports them across the magnetic field, and the drum facilitates the separation process. The performance of BECS heavily depends on the precise synchronization and dynamic adjustment of these components.

Despite their widespread use and theoretical efficiency, traditional BECS units still rely on manual configuration and fixed parameters to control speed, angle, and feed rate. This static tuning often results in suboptimal performance, increased energy consumption, and reduced throughput. Moreover, operational issues such as conveyor belt misalignment, irregular material shape or size, and the presence of ferromagnetic contaminants can degrade separation quality and pose serious risks, including mechanical failure or fire hazards. These challenges become more critical when dealing with heterogeneous and contaminated waste streams, which are standard in industrial environments.

To overcome these limitations, there is a growing demand for intelligent, adaptive systems that can dynamically optimize separation performance. Integrating advanced sensors, AI models, and digital twin technologies into BECS offers a promising solution to enhance operational reliability, improve separation accuracy, enable predictive maintenance, and increase the safety of the entire recycling process. The remainder of this paper presents such a system and its industrial validation.

The primary contributions of this paper are as follows:

- The development of an intelligent optimization model for a conveyor belt, vibration feeder, and magnetic drum angle in a BECS system using real industrial data.
- The integration of a machine vision module with YOLOv11 and a line-scan camera to classify materials by shape (sharp or round).
- Implementation of a thermal monitoring system using an infrared camera to detect fire hazards from ferromagnetic materials.
- A fully automated conveyor belt misalignment correction mechanism based on edge detection and motor control.
- End-to-end integration with a Digital Twin and PLC infrastructure, enabling real-time monitoring, prediction, and self-adjustment.

### 1.1. Background

**Figure 2:**
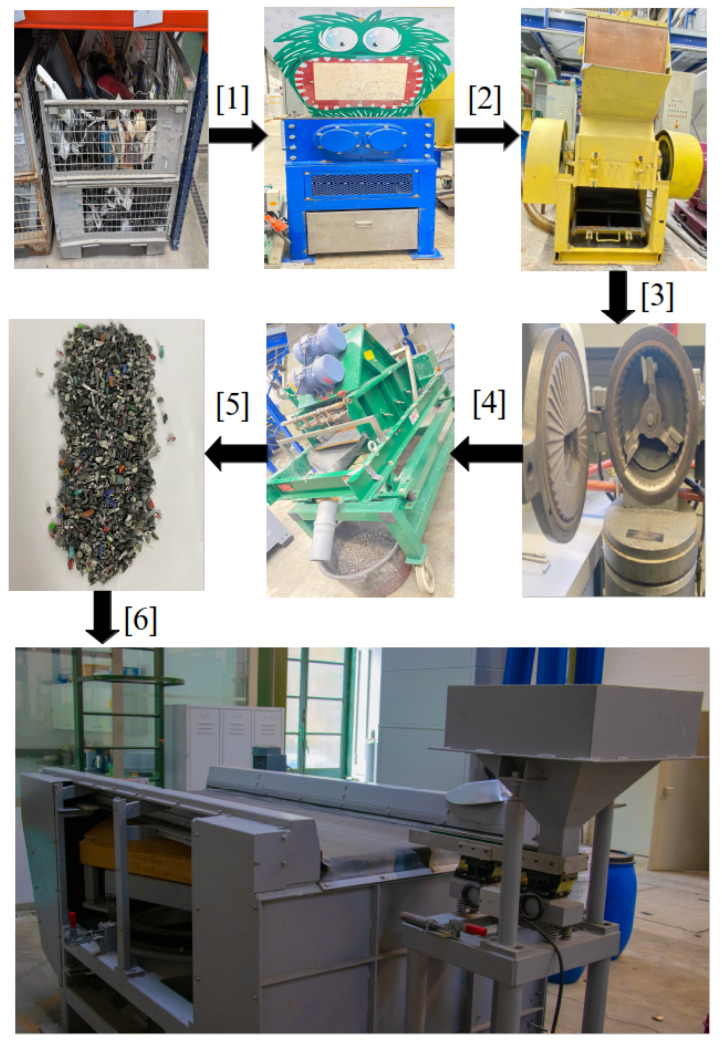
(1) Collected waste electronic components. (2) First shredding machine, used to break down larger components into coarse fragments. (3) Second shredding machine, which further reduces particle size for better separation. (4) Milling machine for smoothing sharp edges of shredded materials. (5) Vibratory screener machine for classifying shredded particles by size before separation. (6) Barrier eddy current separator.

With the rapid advancement of technology and the shortening lifecycle of electronic products, the volume of e-waste has grown dramatically. These discarded devices contain valuable components such as aluminum, copper, brass, and plastics that can be recovered through proper recycling. However, before entering a BECS, the raw materials must undergo several pre-processing steps to meet safety and performance requirements. Figure 2 illustrates the process of preparing materials for entry into the eddy current separator step by step.

In the first step (1), old and discarded electrical and electronic devices such as computer cases, wires, plastic, and metal parts are collected in large storage cages. These materials consist of a mixture of plastics, non-ferrous metals such as aluminum and copper, and other components that must be processed before effective separation can occur. In the second step (2), the collected devices are fed into a shredder. This initial shredding machine uses powerful blades to break down the large items into smaller, more manageable pieces suitable for further grinding. In the third step (3), the pre-shredded materials are passed through an industrial granulator, which further reduces the particle size to a medium range, preparing them for fine grinding.

The fourth step (4) involves the delicate grinding process, where the materials enter a precision mill grinder. This machine reduces the particle size to approximately 1 to 4 mm, which is essential for the optimal performance of a BECS. The separation process becomes less effective if the particles are too large or too fine. The fifth step (5) transfers the ground materials to a vibrating sieve. This sieve sorts the particles by size, ensuring that only particles within the desired range (1 to 4 mm) proceed to the next stage. Finally, in step six (6), the prepared mixture containing non-ferrous metals and plastics is fed into the eddy current separator.

A BECS, using a rapidly rotating magnetic field, separates non-ferrous metals like aluminum and copper from non-metallic materials such as plastics, completing the final stage of the recycling process.

### 1.2. Issues

Despite their key role in non-ferrous metal recycling, traditional BECS systems face several challenges. Conveyor misalignment, surface wear, and tear, and the presence of ferromagnetic or sharp-edged materials can lead to poor separation, mechanical failure, or even fire near the magnetic drum. The manual control of components, such as conveyor speed and drum power, makes the system susceptible to delays and human error, particularly in emergency situations. Additionally, running the system without material input wastes energy and accelerates wear and tear. Separation errors are frequent due to the lack of intelligent feedback, leading to material recirculation, energy waste, and operator fatigue. Continuous 24/7 operation further strains human resources, with high costs and increased error rates, especially during night shifts. The harsh recycling environment also poses health and safety concerns.

These limitations highlight the need for an intelligent BECS system that integrates machine vision, real-time sensing, PLC automation, and digital twin technologies to enhance safety, efficiency, and reliability while reducing human dependency.

### 1.3. Existing Body of Knowledge and State of the Art

Recent advances in material recycling, especially plastic separation, have increasingly leveraged AI, machine vision, and robotics. For instance, ref. [9] explores a human-centered robotic system that utilizes machine vision, deep learning, and FTIR spectroscopy to enhance separation accuracy and sustainability while still involving human operators. Similarly, ref. [10] integrates optical spectroscopy with algorithms such as CNN, YOLO, and random forest to address challenges, including dark or composite plastics, thereby offering a broader spectroscopic and classification scope.

Practical implementations are highlighted in [11,12], where robots and YOLO-based models classify materials such as PET, glass, and metal bottles using RGB, NIR, and VIS sensors. These studies mark a shift from conceptual models to real-world deployment. On a systemic scale, ref. [13] envisions the integration of IoT, blockchain, and AI throughout complete waste management cycles, from prediction to route optimization, while addressing policy and privacy concerns.

Together, these works outline a clear research path: from precise material detection [9,10] to industrial automation [11,12], and ultimately, to broader innovative waste systems [13], all aiming to improve efficiency, reduce errors, and support a circular economy.

Much work has focused on optimizing BECS design in non-ferrous metal separation. Particle size is a key factor, as shown in [14,15], where very small or large particles reduce efficiency due to their low magnetic response or current disruption. Magnetic roller design and pole configuration are, therefore, crucial.

Modeling also plays a key role: ref. [7] developed a differential-equation-based model to predict particle motion in magnetic fields, showing accuracy for various shapes except spheres. Ref. [6] reviews ECS principles and challenges, particularly with particles under 5 mm, while highlighting design limits and progress. Environmental perspectives are covered in [8], which compares ECS designs and presents them as sustainable, low-pollution alternatives for e-waste recycling. Collectively, refs. [6,7,8,14,15] offer a comprehensive view of BECS development, from modeling and design to environmental impact, underscoring the need to balance technical performance with sustainability.

### 1.4. Gap Detection

A comprehensive review of recent publications in the fields of material separation and industrial recycling reveals that, despite considerable advancements in machine learning, computer vision, industrial automation, BECS design, and the Internet of Things, several key gaps remain in the development of a fully autonomous and intelligent BECS system. Most prior studies have focused on individual subsystems within BECS rather than addressing the system as an integrated whole. For instance, studies such as [6,7,14,15] concentrated on magnetic drum design or the dynamic behavior of particles. However, the interaction and synchronization between the conveyor belt, vibration feeder, and magnetic drum under real industrial conditions remain underexplored. The particle behavior models developed in these works are generally based on simulations with idealized assumptions rather than practical deployments.

At the same time, although the concept of digital twins has been extensively explored in domains such as energy, mining, and chemical processes [16,17,18,19,20], no real-world implementation has yet been documented in the context of ECS or BECS systems for electronic waste recycling. In the domain of computer vision, most works such as [9,10,11,12] have focused on classification based on material color or type. Meanwhile, studies such as [21,22] have addressed edge or surface defect detection in industrial settings but have generally been limited to flat or large-scale components. No prior study has specifically addressed the detection of the physical shape (sharp vs. round) of fine, powder-like conductive particles (1–4 mm in size) within dusty ECS environments, even though sharp particles pose serious risks to conveyor integrity, safety, and separation performance.

Although research such as [23,24] acknowledges the importance of thermal and magnetic monitoring systems in recycling facilities, these systems are typically implemented in isolation, lacking meaningful integration with central industrial control frameworks such as AI, IoT, and PLCs. No documented example of a linkage between thermal hazard detection and immediate industrial actuator response exists in BECS systems.

Rapid growth in IoT-based solutions and control, as seen in studies such as [25,26,27], has led to the development of Raspberry Pi-based lightweight monitoring systems, as described in [28]. These have enabled basic data acquisition and local classification. However, none have successfully proposed a multi-layer, reactive architecture that fully integrates the data acquisition, material recognition, decision logic, and automated execution in BECS operations. Similarly, the use of PLCs, as reported in works such as [29,30,31], is often confined to simple, rule-based control schemes. Even in cases involving predictive maintenance and failure analysis [32,33], most approaches remain offline and descriptive, lacking the response capacity.

### 1.5. Research Questions

**RQ1:** How can unwanted incidents on the conveyor belt in industrial separator systems be prevented?

**RQ2:** How can the separation of metallic and non-metallic materials be enhanced in a barrier eddy current separators while maintaining a high input throughput?

**RQ3:** How can identifying the type of materials on the conveyor belt contribute to improving the separation quality in an eddy current separator?

This work proposes a fully integrated, intelligent BECS system that combines machine vision, thermal monitoring, machine learning, and industrial control to address the limitations identified in previous research and practical implementations. Unlike earlier approaches focused on isolated subsystems, our solution offers a holistic architecture in which all three critical components—conveyor belt, vibration feeder, and magnetic drum—are dynamically optimized through a data-driven multi-output regression model. This model, trained on real industrial datasets, suggests the most efficient speed and angle configurations based on input conditions, thereby enhancing the separation quality, reducing energy consumption, and minimizing mechanical stress.

Moreover, the system incorporates the concept of a digital twin that simulates the physical BECS by using live sensor and camera data. This enables predictive analysis, fault detection, and performance monitoring without interrupting operations. The machine vision module utilizes a set of cameras and a YOLOv11-based deep learning model to detect the shape of fine particles (sharp vs. round), which is crucial for conveyor integrity and separation accuracy—an aspect previously overlooked in studies. Additionally, the thermal camera detects the overheating caused by ferromagnetic contaminants and triggers a direct PLC response to halt the system, thereby preventing fire hazards. Through the seamless integration of intelligent perception, decision making, and autonomous actuation, our system bridges the gap between theoretical modeling and real-world industrial demands, providing a scalable and resilient solution for modern recycling environments.

The paper is structured as follows: Section 1 provides an overview of the research background and outlines the key research questions motivating this work. Following this, Section 2 reviews the supplementary literature and related studies that form the foundation of the proposed system. Building upon these insights, Section 3 presents a detailed description of the overall system architecture and its modular design. Subsequently, Section 4 elaborates on the implementation of the core modules, including shape detection, speed optimization, fire monitoring, and belt alignment. Section 5 then discusses the system testing process and presents the evaluation results. Finally, Section 6 summarizes the main findings and outlines potential directions for future research.

## 2. Supplementary Literature and Related Work

A comprehensive review of the literature on the intelligent optimization of material separation systems reveals substantial progress in machine vision, machine learning, industrial controllers, the Internet of Things (IoT), and digital twin technology. However, most studies focus on narrow aspects and fall short of providing a deployable, integrated solution for eddy current separation (ECS) systems, especially under real industrial conditions, such as those encountered by barrier ECS (BECS) units. Our project, Twin-AI BECS, is designed to bridge this gap by combining machine vision, AI-based optimization, thermal monitoring, and digital twin infrastructure into a single operational platform.

Studies such as [9,10] have introduced robotic systems leveraging machine vision, deep learning, and spectroscopy for plastic classification. Although promising, these models are typically deployed in structured or household environments and fail to address the complexities of industrial contexts involving fine metal powders. Twin-AI BECS tackles this challenge by integrating YOLOv11 with a line-scan camera to classify the shape of fine metallic particles (1–4 mm) under dusty and vibrating conditions.

Research by [11,12] explored robotic separation with sophisticated mechanical components and multi-sensor arrays. While effective, their high cost and complexity hinder scalability. In contrast, our solution utilizes cost-effective Raspberry Pi units, industrial-grade cameras, and lightweight AI models for practical deployment across BECS lines.

From a systems perspective, ref. [13] emphasizes macro-level IoT and blockchain systems for waste logistics and incentive-driven recycling. Twin-AI BECS instead focuses on micro-level optimization and direct machine feedback using onboard sensors, fusion modules, and autonomous actuation. Physical modeling studies, such as cite bin2022effects, shan2024effects, smith2019eddy, rem1997model, provide mathematical insights into magnetic roller effects and particle motion under ideal laboratory conditions. However, they do not consider hardware constraints, dust accumulation, or real-time dynamics. Our system complements these works by utilizing real-world data from 82 test cases to train regression models that optimize conveyor speed, vibration intensity, and drum angle in real-time.

Digital Twin research, while evolving rapidly, remains underutilized in the ECS domain. Frameworks in [16,17,18,19,20] propose general simulation or monitoring systems that do not require deployment in ECS machinery. Our work represents the first real-world deployment of a Digital Twin for BECS, featuring PLC integration, simulation capabilities, fault detection, and predictive decision support.

Industrial control studies [29,30,31,34] mostly rely on rule-based ladder logic, lacking AI or vision feedback. Similarly, works such as [23,24,35] discuss thermal or fire detection as isolated safety mechanisms. In contrast, our system utilizes thermal imaging to monitor critical areas and automatically halt operation through the PLC if overheating is detected, thereby enhancing both equipment safety and worker protection. Lightweight controller designs based on Raspberry Pi and PLC, such as [25,28,36,37], often remain limited to lab-scale implementations with no feedback-based actuation. Our approach extends this by enabling the PLC-controlled real-time decision-making and deploying robust YOLO-based vision analysis.

Additionally, modeling-focused efforts, such as [38,39,40], concentrate on magnetic force computation but disregard real-world complications, including belt misalignment, particulate contamination, or heat hazards. We bridge this gap by integrating real-time monitoring, digital twin modeling, and vision-based classification in a rugged industrial prototype.

A remarkably underexplored area—vision-based conveyor belt fault detection—is examined in [21,41,42], where surface issues are detected, but corrective mechanisms are absent. Our project advances this domain by automating belt misalignment correction using a line-scan camera and a dual-stepper system.

Finally, blockchain- and incentive-based recycling strategies, such as [26,43], focus on human behavior rather than industrial feasibility. Moreover, although platforms in [27,44] propose intelligent robotic architectures and scheduling algorithms, their mechanical complexity makes them incompatible with ECS constraints. Twin-AI BECS delivers a realistic, industrially deployable solution that balances modularity, cost-efficiency, and intelligent automation.

In the paper [45], the authors propose a mixed-effects multivariate degradation framework to disentangle fixed design effects from unit-to-unit variability using accelerated degradation data. The model aims to assist in reliability-based design decisions for complex electronic systems by isolating the impact of design configurations from manufacturing heterogeneity. While this work provides valuable statistical insights into long-term durability and informs design selection, our approach differs fundamentally in scope and application. Their focus lies in reliability prediction and design optimization through the statistical modeling of degradation processes. In contrast, our system targets the real-world deployment of an integrated BECS platform, combining eddy–current separation, real-time machine vision (YOLOv11), digital twin modeling, and industrial sensor infrastructure. Our contributions extend beyond theoretical modeling: we provide quantitative performance metrics collected from operational environments, including 92% edge detection accuracy, mAP@0.5 exceeding 0.9, and robust real-time safety and sorting performance. Unlike the statistical framework, our platform demonstrates not only theoretical validity but also practical effectiveness in live industrial conditions, reinforcing its superiority and immediate applicability over prior research.

## 3. System Overview and System Architecture

As outlined in Section 1 and Section 2, existing research focuses on selectively integrating sensors and more or less sophisticated analysis approaches into recycling systems for waste stream separation. To integrate not only one but a scalable number of smart services to address the detected gap in Section 1.4, a modular, scalable, and adaptable system architecture is required.

**Figure 3:**
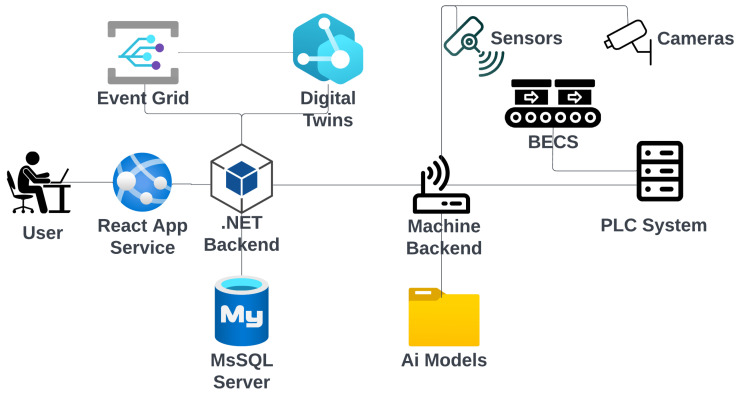
High-level system overview.

Figure 3 presents a high-level perspective of the systems overview. The system’s core is a .NET backend cloud application that can manage a multitude of BECS machines in various locations, each managed by a separate machine backend. Subsequent AI models are also deployed locally on the machine’s backend and use camera and sensor inputs to control each BECS via its PLC. A digital twin and its corresponding event grid provide a digital representation of each BEC based on information from the machine backend received via the .NET backend cloud application. Users interact with the system through a React service app connected to the .NET cloud backend.

**Figure 4:**
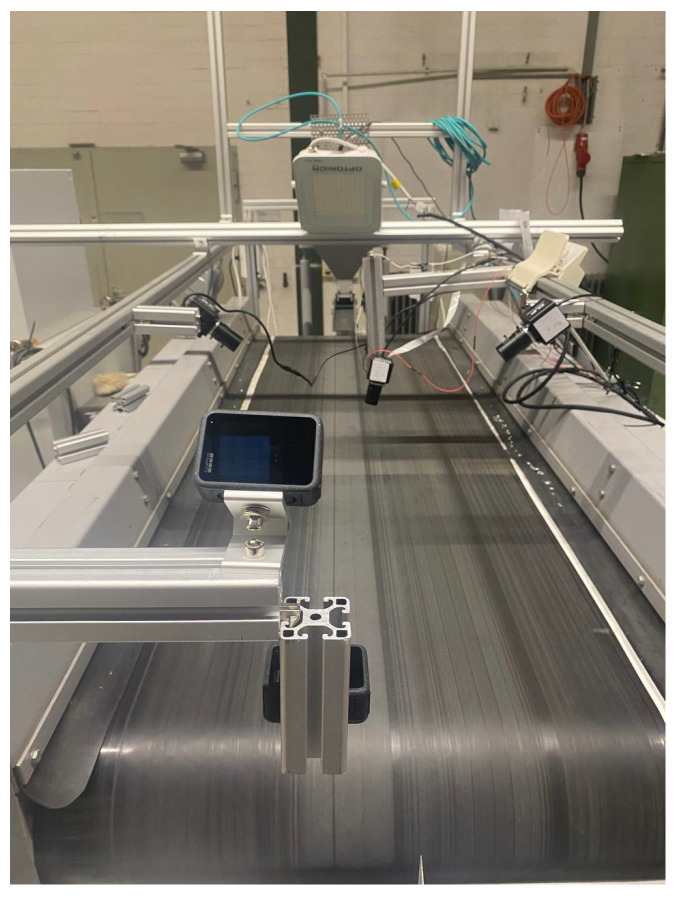
Camera setup with multiple devices (ELP, Raspberry Pi camera, GoPro) used for dataset collection.

Subsequent Figure 4 presents aspects of the camera setup. A frame is constructed over the BECS conveyor belt, and different cameras (four ELP 8 Megapixel Webcam with HD 5–50 mm cameras, one Raspberry Pi camera, two GoPro Hero 12 cameras, a Basler raL 12288-8gm1 line-scan camera, and one MLX90640 thermal camera). The cameras, in conjunction with additional sensors, are used for initial data acquisition as well as constant monitoring of the BECS operation, material on the conveyor belt as well and occurring undesired events, e.g., too much or too little material on the belt, wrong material on the belt, fire hazards, etc.

Subsequently, Section 3.1 details the system architecture and design, while Section 3.2 introduces our digital twin concept. Afterward, Section 3.3 focuses on the PLC system integration.

### 3.1. System Architecture

**Figure 5:**
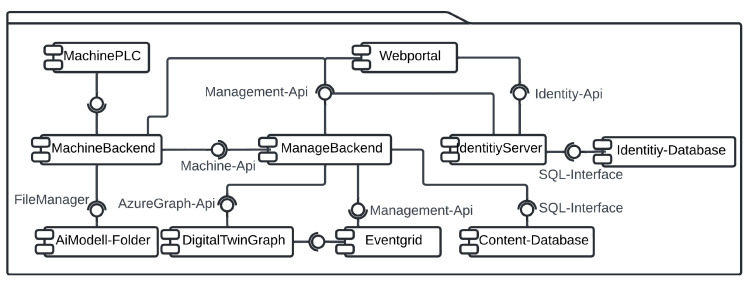
High-level system architecture.

**Figure 6:**
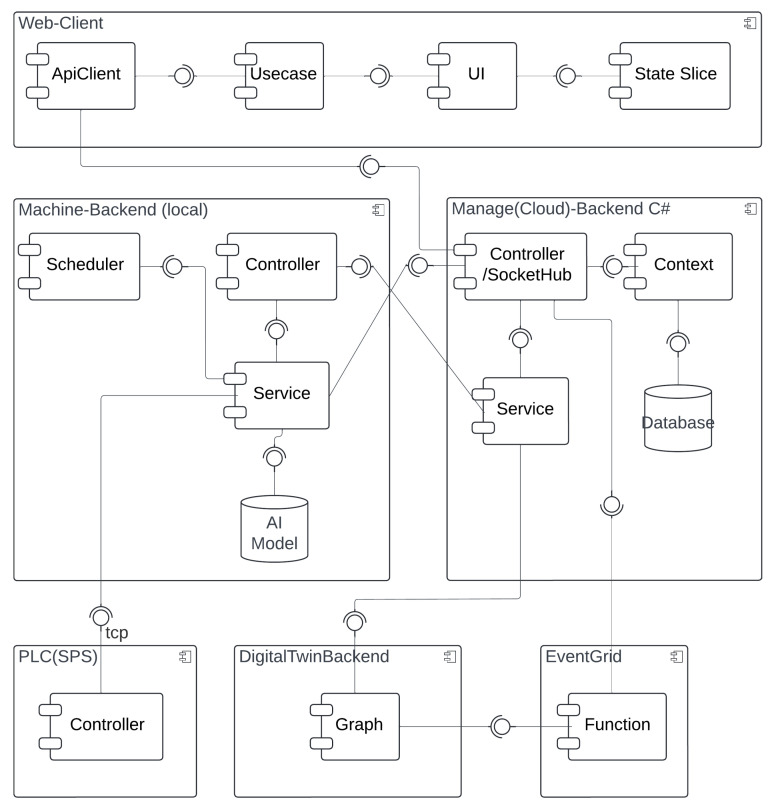
Overview high-level architecture.

Figure 5 and Figure 6 illustrate the high-level system architecture as well as the respective refinement of the BECS control platform, which is implemented as a set of loosely coupled micro-services that communicate exclusively via well-defined http, WebSocket and event interfaces. The user-facing **Web Portal** (React) constitutes the single entry point for operators, whereas two backend tiers enforce a strict separation between cloud-level coordination (**ManageBackend**) and shop-floor execution (**MachineBackend**). Domain-independent concerns such as authentication and authorization are delegated to an external **IdentityServer** (Duende, oidc-compliant); persistent machine and content data are stored in dedicated SQL databases, and time-varying telemetry is modeled in an **AzureDigitalTwins** graph that is updated and observed via **EventGrid**. A **ProgrammableLogicController** (plc) provides real-time access to the physical separator and is abstracted by a lightweight **MachinePLC**-driver.

**WebPortal (React):**  Each ui component manages its data and directly invokes *Use-case* modules, ensuring clear functional boundaries and high testability. Cross-component dependencies are handled by lightweight *state-slices* that broadcast mutation events without storing domain data, thereby avoiding the complexity of a global store while guaranteeing coherence.
**ManagementBackend (.net 7):**  A minimalist controller/hub layer accepts rest and WebSocket traffic; a thin service layer encapsulates integrations with Azure Digital Twins and Event Grid; and ef Core’s DbContext offers transactional access to the structured content database. This layered approach decouples business logic from transport concerns and allows horizontal scaling behind an Azure App Service during peak throughput.
**MachineBackend (Python/Flask):**  Deployed at the edge, it augments raw sensor data with ai-based inference (YOLOv11 object detection, anomaly scoring) before relaying only high-value information northbound. A built-in scheduler executes periodic acquisition and model-evaluation tasks locally, reducing cloud round-trips and guaranteeing the deterministic response times required by the BECS.
**DigitalTwin and Event Grid:**  The graph model provides a semantically rich, versioned representation of conveyors, drums, and feeders, facilitating *what-if* simulations and complex-event detection. Event Grid’s serverless pub/sub pattern decouples producers and consumers, so new analytic modules can be attached without modifying the existing code.
**IdentityServer:**  Outsourcing authentication to an oidc-compliant Duende service eliminates the duplicated security code across micro-services and enables seamless single-sign-on for the web portal, mobile clients, and future api consumers.

A typical optimization cycle begins when an operator requests a new conveyor speed through the WebPortal. The ui component triggers a *Use-Case* function that issues an authenticated http call to the ManageBackend. The request is routed to the MachineBackend and finally forwarded over tcp to the plc. Once the plc confirms the set-point change, the MachineBackend publishes the new state to the ManageBackend, which persists in the sampling, updates the corresponding Digital Twin node, and emits a change event via EventGrid. The ManageBackend receives that event, pulls the updated graph snapshot, and pushes the fresh data to all subscribed browser sessions via a WebSocket hub, thus providing the operator with instantaneous visual feedback.

**Figure 7:**
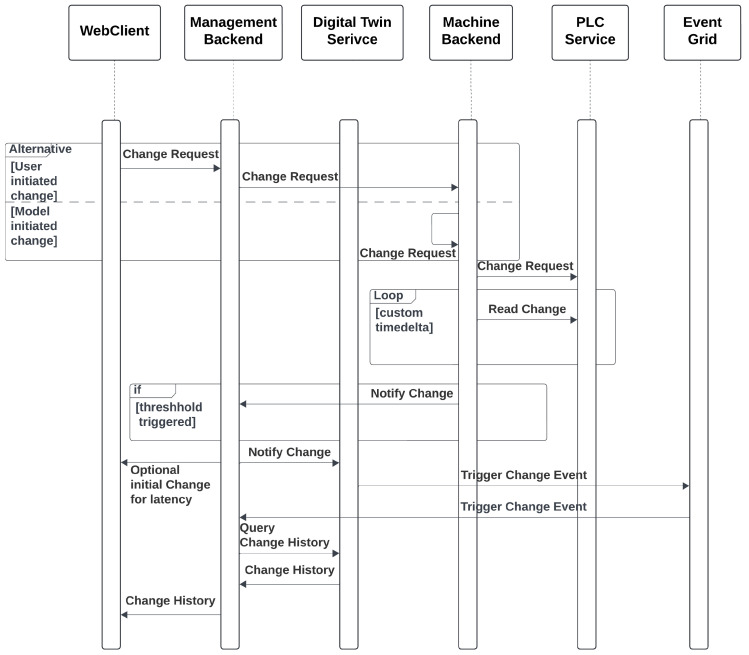
Request flow of change events.

In addition to operator-triggered adjustments, the system also supports *autonomous optimizations* initiated by local AI models deployed within the MachineBackend. When the embedded inference module—executing tasks such as throughput prediction or anomaly detection—determines that the conveyor speed should be modified (e.g., due to a sudden change in material density), it directly issues a set-point update to the plc via its internal driver. Upon successful actuation, the new speed is published to the ManagementBackend in the same format used for operator interventions. From this point forward, the update follows the established propagation path: The ManagementBackend persists the new state, updates the affected digital twin node, and emits a corresponding event through EventGrid. This guarantees that all subscribed clients, including the WebPortal, receive a consistent and timely view of the system state—regardless of whether changes were human- or machine-initiated. The corresponding process flow is illustrated in Figure 7.

While this proof-of-concept omits formal data validation and failure handling, state updates are only pushed when the physical device reports a change that surpasses a predefined threshold, which is hardcoded in the MachineBackend. Consequently, the frequency of updates is event-driven and depends on the dynamic behavior of the material stream. The end-to-end latency—from the moment a physical change is detected by the sensor, through inference, actuation, propagation, and finally visualization in the browser—varies significantly depending on the deployment environment. In a well-provisioned industrial setup with edge compute resources located physically close to the shop floor and a low-latency cloud backbone (e.g., Azure Germany Central), round-trip latencies on the order of 200–500 ms are feasible, especially if the WebPortal gets notified from the ManagementBackend directly and not via the EventGrid roundtrip. However, in less optimized deployments—such as when using shared cloud infrastructure, remote telemetry links, or under high load—latencies may extend into the 1–2 s range or beyond. These variances stem from the cumulative effect of several asynchronous components (e.g., WebSocket hubs, EventGrid propagation, Digital Twin updates) and underline the importance of tailored infrastructure design in latency-sensitive industrial applications. At present, no versioning or rollback mechanisms are implemented; however, every property update is recorded in the time-variant store of the digital twin graph, enabling retrospective analysis and the historical correlation of control decisions.

Collectively, these design decisions yield a platform that is *modular* (services can be replaced independently), *observable* (every state change is mirrored in the digital twin), and *autonomous* (closed-loop control via plc integration). The resulting architecture fulfills the stringent real-time and reliability requirements of industrial recycling environments while remaining flexible enough to host advanced optimization and safety modules in future work.

### 3.2. Digital Twin

A digital twin is a bidirectionally coupled cyber-physical model that reproduces, with minimal latency, the evolving state of a particular asset, process, or socio-technical system. The approach combines physics-based simulations, statistical system identification, and continuously streaming sensor data to establish a closed feedback loop between the virtual representation and its physical counterpart. While the early applications of the digital twin concept emerged in aerospace [46] and manufacturing [47], it has since been utilized in various fields, including food production [48], the automotive sector [49], Industry 4.0 [50], and others.

**Figure 8:**
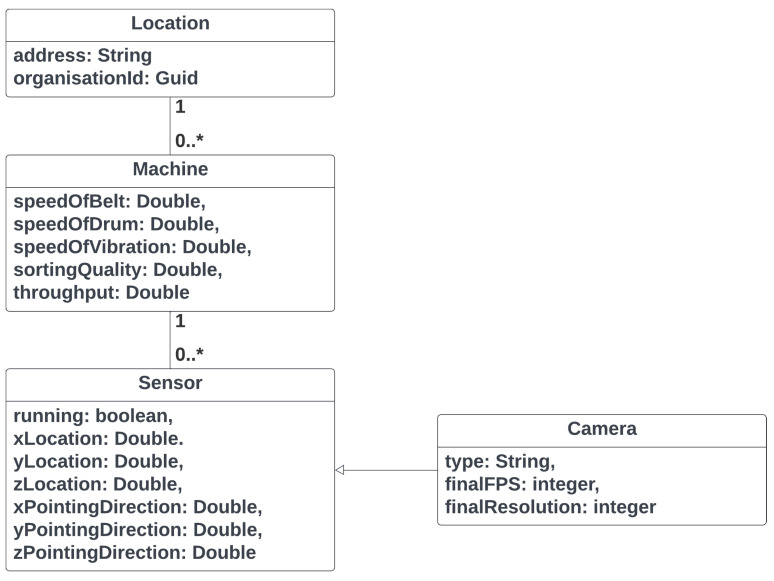
Digital Twin—class diagram.

**Figure 9:**
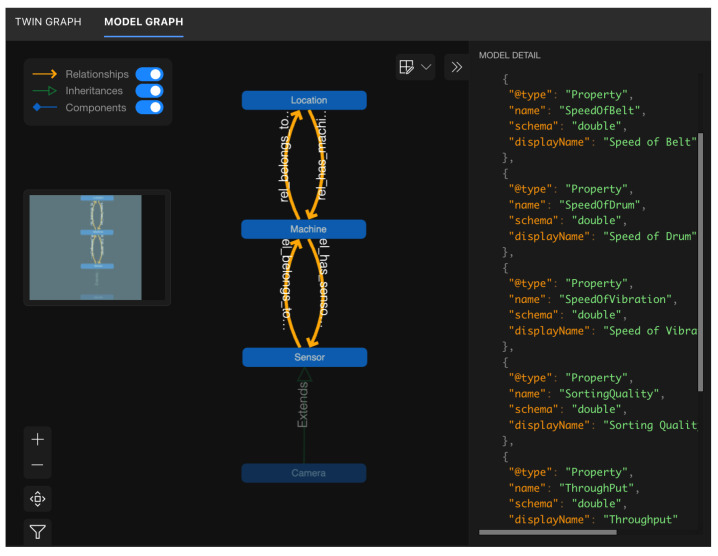
Digital Twin—exemplary data perspective.

Azure Digital Twins (ADT) was selected because it natively offers (i) a *graph-oriented* data model that mirrors the physical hierarchy of recycling plants, (ii) a *time-variant property store* that can be queried with millisecond latency, and (iii) an event stream that integrates seamlessly with the platform’s serverless services (Event Grid, Functions). These characteristics align with the requirements of the BECS scenario, where individual machines must be contextualized within specific locations, sensors, and higher-level organizational units, while every property change (e.g., belt speed, drum angle) must be propagated to analytic and visualization components in near real-time. Alternative solutions—such as storing telemetry in a relational schema or blob storage—would have demanded additional middleware to capture topology information and to emit change notifications, thereby increasing complexity and maintenance effort. Figure 8 presents a simplified class diagram of the used digital twin model, while Figure 9 illustrates an example of a digital twin for a BECS system in operation.

**Domain model:**  The logical structure of the plant is expressed in *Digital Twins Definition Language* (DTDL). Location aggregates any number of Machine twins via the rel_has_machines relationship; each Machine twin exposes operational properties such as SpeedOfBelt or SortingQuality and maintains bi-directional links to its Sensor set. Specialized sensors—e.g., a line-scan Camera—inherit from the generic sensor interface to avoid duplication. This inheritance pattern allows us to extend the model (e.g., add a hyperspectral camera) without refactoring existing code.
**Service integration:**  An ADT instance is provisioned in the same Azure region as the Manage Backend to minimize latency. The ManageBackend exposes a thin TwinService that wraps the Azure.DigitalTwins.Client SDK: the service updates and inserts property patches received from the MachineBackend and establishes relationships on first contact. All twin modifications are configured to trigger an EventGrid topic, which disseminates messages to a function that stores snapshots in cold storage for offline analytics, as well as to the WebSocket hub that feeds the operator dashboard.
**Outcome:**  By adopting ADTs we achieved a single source of truth for both the *static* topology and the *dynamic* state of the BECS. The graph query language has simplified cross-asset correlations (e.g., which sensors are attached to machines in location A?), while the built-in eventing mechanism reduced round-trip latency between PLC state changes and browser visualization.

### 3.3. PLC System Integration

The system presented in this study is a modernization of an existing BECS used in recycling processes. The original control system was replaced by a modern Siemens S7 PLC, which offers a flexible and scalable automation platform. The upgrade aimed to significantly improve both the control precision and the system’s adaptability to different material compositions. In addition to the PLC upgrade, the system was equipped with a comprehensive range of sensors, high-resolution video cameras, and line-scan cameras, as outlined in previous sections. These components continuously monitor and record data from the material flow and provide a detailed insight into the separation process. The recorded data is streamed via the Internet to a digital twin of the system, which virtually displays the operating status in real-time on a web-based digital twin platform developed in-house.

The digital twin not only serves as a monitoring and diagnostic tool but also as a data source for training machine learning models. These models are designed to analyze patterns in process behavior and predict optimal control settings. After training, the ML algorithms operate in near real-time to dynamically adjust the most critical control and regulation parameters of the eddy current separator via the PLC. This integration of classic industrial automation with data-driven optimization enables a responsive, self-adapting system. The result is improved sorting accuracy, increased system efficiency, and reduced manual intervention.

**Figure 10:**
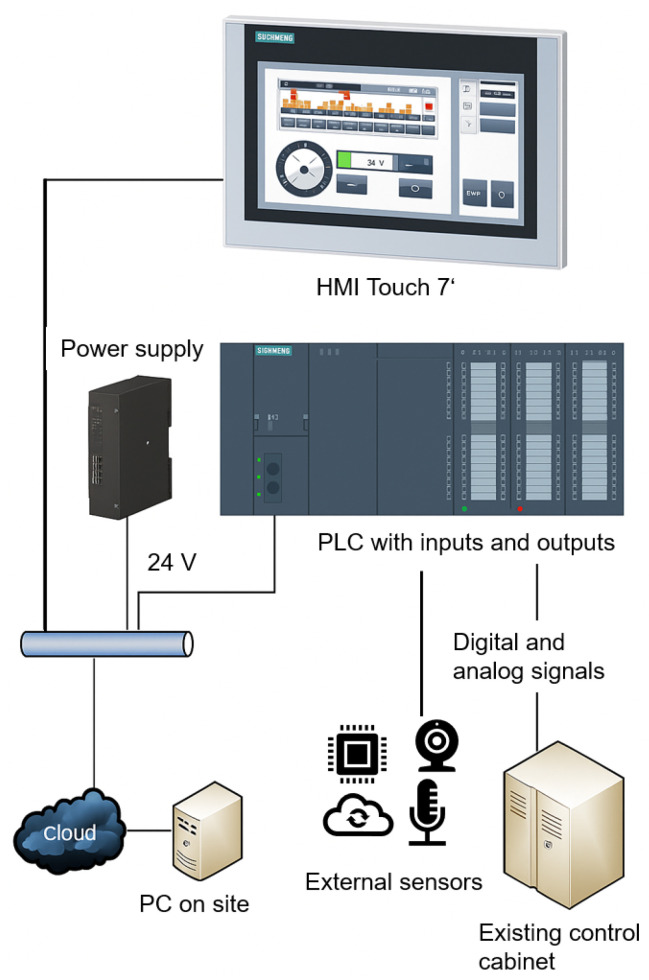
PLC system integration.

The basic structure of the PLC system integration is shown in Figure 10. The central element is the BECS, which features an integrated PLC. Digital and analog signals from the existing control system are recorded via various inputs and outputs, and modern external cameras and sensors are also newly integrated. The PLC implemented with a Siemens S7-1500 communicates with all peripheral devices via PROFINET or OPC UA and executes a structured control logic developed in TIA Portal. The control program handles data acquisition in real-time, actuator control, interlocks, and process sequence control. An HMI touch interface enables live system monitoring, user interaction, and diagnostics.

## 4. Implementation of Core Modules

This project integrates AI, computer vision, and IoT modules to enhance BECS performance in real industrial settings. Key functions include material presence detection, type and edge classification, belt misalignment correction, fire prevention via thermal imaging, and ML-based parameter optimization—improving accuracy, safety, and efficiency.

### 4.1. Color-Based Material Detection on Moving Components

**Figure 11:**
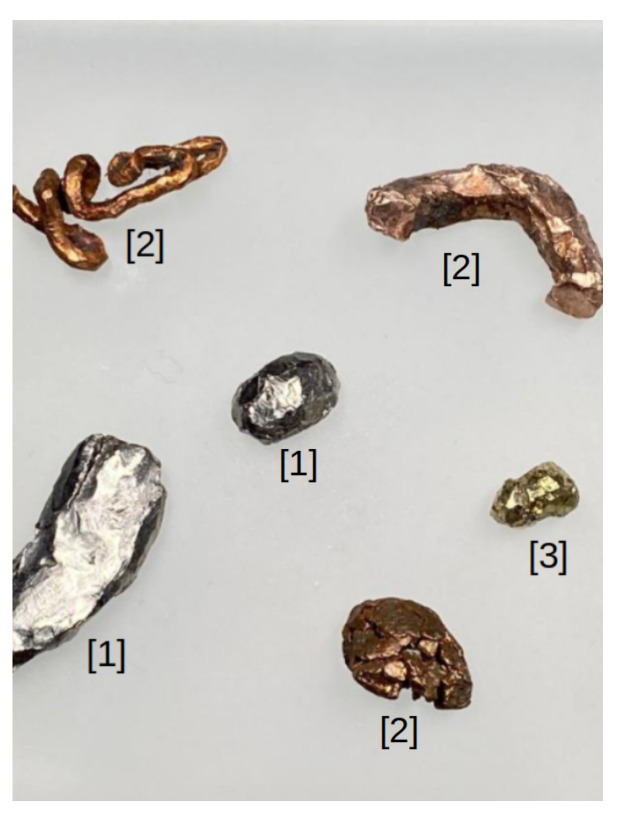
(1): Aluminum; (2): copper; and (3): brass.

Material presence detection and color-based classification are implemented on both the vibration feeder and the conveyor belt, utilizing a combination of traditional computer vision and deep learning methods to enhance material classification and minimize energy consumption in the separation system. On the vibration feeder, early system versions showed that the conveyor often ran empty for long periods, consuming unnecessary energy while minimizing throughput. To address this, a motion sensor is installed to detect the presence of materials before initiating processing. Once material presence is confirmed, the system performs color-based classification using OpenCV [51], identifying aluminum, copper, and brass particles which are illustrated in Figure 11. This two-step approach enhances the processing efficiency and classification accuracy.

**Figure 12:**
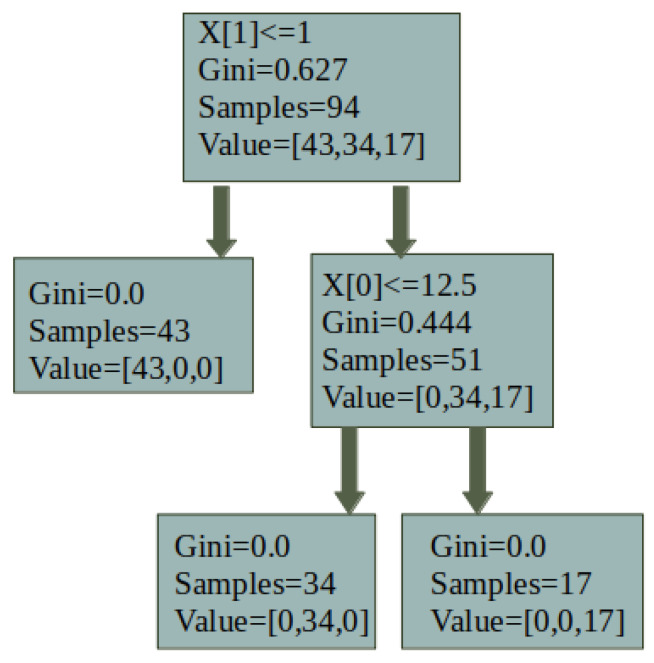
Structure of the decision tree.

**Figure 13:**
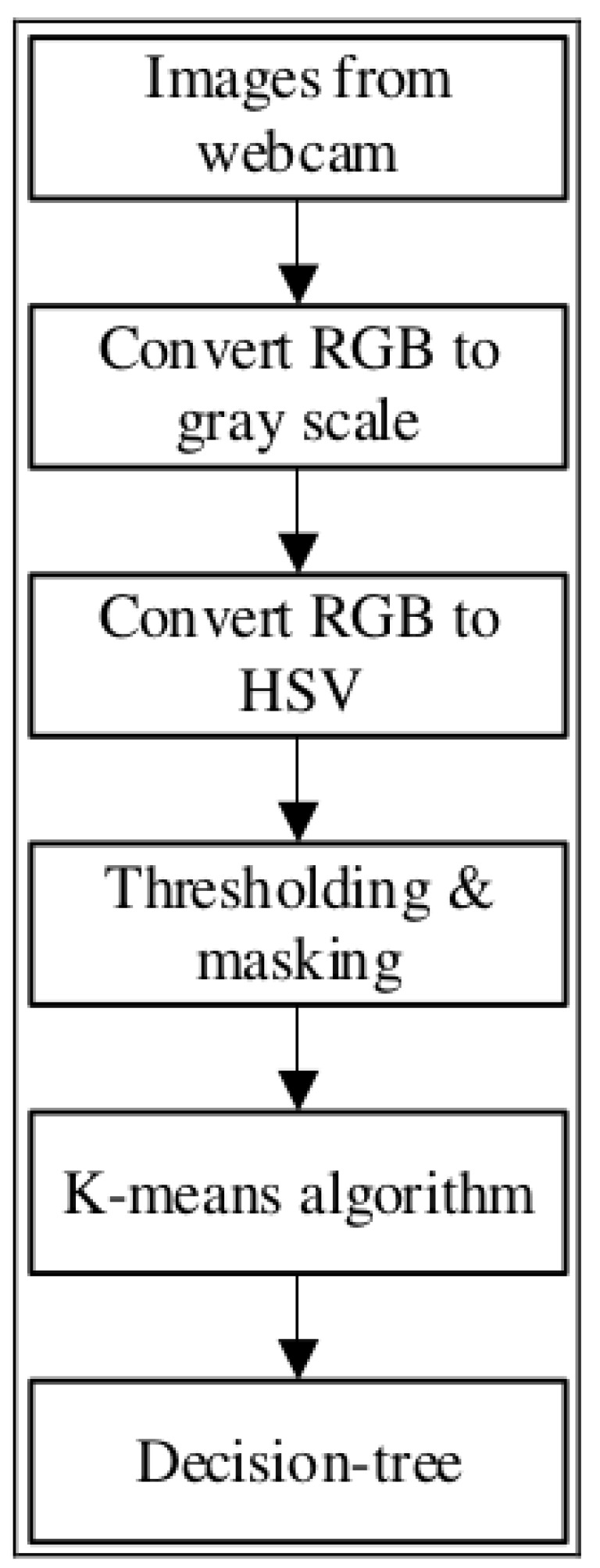
Model process chart.

An ELP camera and motion sensor are mounted above the vibration feeder to improve the recognition under noisy industrial conditions. Motion sensors are used for motion detection, and OpenCV functions are used for texture analysis, material counting, and feature extraction. Machine learning algorithms, such as K-means and decision trees, are applied to enhance robustness. A decision tree is useful for materials with overlapping colors, leveraging dominant color ratios (e.g., >80% gray for aluminum, orange for copper). This two-step approach enhances processing efficiency and classification accuracy (see Figure 12 and Figure 13). The decision tree logic, based on the Gini index, improves classification precision:

(1) $$Gini=1-\sum_{i=1}^{c}{\left(P_{i}\right)}^{2}$$

**Figure 14:**
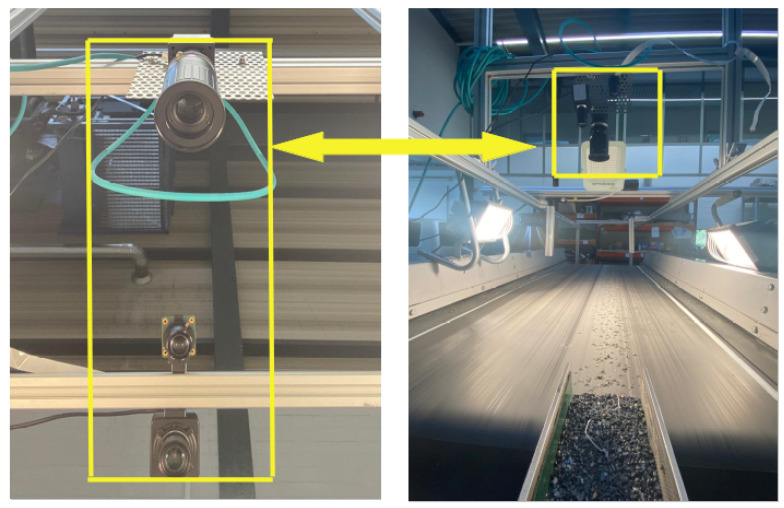
Camera setup for material inspection on the conveyor belt of the barrier eddy current separator machine. The right image shows the top view of the conveyor belt with the mounted line-scan camera, Raspberry Pi camera ELP camera and the lighting system. In contrast, the left image provides a close-up view of the vertically aligned cameras.

A more advanced setup was needed on the conveyor belt to classify high-speed material flow under varying lighting conditions. As illustrated in Figure 14, the system uses multiple cameras (line-scan, ELP, and Raspberry Pi) mounted above the belt. Images are collected under various lighting conditions and viewing angles to simulate industrial environments and subsequently processed using the Roboflow platform for annotation and segmentation.

Due to its robustness in detecting small objects in motion, the YOLOv11 model is employed to automate classification on the moving conveyor. YOLOv11 outperforms other tested models, accurately classifying aluminum, copper, brass, and plastics across various conveyor speeds and lighting conditions. The model’s convolutional neural network (CNN) architecture enables simultaneous object detection, classification, and segmentation, ensuring high-speed and high-precision operation.

### 4.2. Preventing Undesired Events in BECS Operation

In real-world BECS operation, three critical risks threaten system integrity and human safety: fire hazards caused by ferromagnetic particles and damage to conveyor belts due to sharp-edged materials and their misalignment. This section outlines the detection strategies developed to prevent such events.

#### 4.2.1. Detection and Correction of Conveyor Belt Misalignment

**Figure 15:**
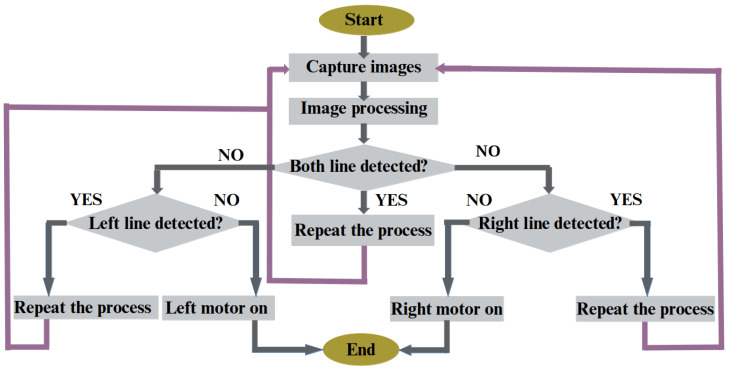
Algorithm process chart.

In the next phase, a machine vision-based system is developed to detect and automatically correct conveyor belt misalignment using a Raspberry Pi camera and two stepper motors. Canny Edge Detection and Hough Transform [52,53,54] detect white lines on the belt. Figure 15 shows the operational flowchart. When one of the sidelines is undetected, the corresponding motor adjusts the belt to recenter it.

Two optimization techniques are applied to enhance performance: Gaussian blur and region of interest (ROI) [55,56,57]. Gaussian blur reduces noise and improves edge detection:

$$G\left(x,y\right)=\frac{1}{2\pi \sigma^{2}}\exp\left(-\frac{x^{2}+y^{2}}{2\sigma^{2}}\right)$$

**Figure 16:**
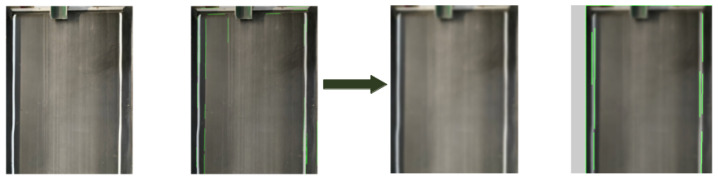
ROI processing steps before and after applying Gaussian blur.

ROI limits image processing to essential conveyor areas, reducing latency and boosting precision. Figure 16 shows the ROI before and after applying Gaussian blur.

#### 4.2.2. Early Fire Detection

**Figure 17:**
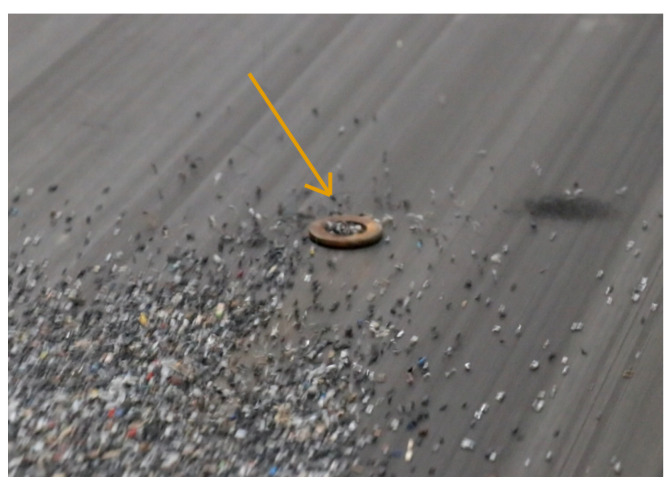
A trapped iron object causing particle clustering and fire risk near the magnetic drum.

Although BECS systems are effective at separating non-ferrous metals, they cannot handle ferromagnetic materials, such as iron. These particles, if accidentally introduced, pose a significant risk of fire. As shown in Figure 17, even a small iron object (orange arrow) can become trapped by the magnetic drum. Instead of being expelled like non-ferrous metals, they remain fixed, accumulate more particles, and generate heat due to friction and magnetic force. Undetected, they may lead to local overheating, smoldering, or even ignition.

To prevent this, a thermal camera-based IoT system is implemented [58,59,60]. The camera continuously monitors the belt temperature, and OpenCV [61,62,63] is used for pre-processing (grayscale conversion, Gaussian blur, adaptive thresholding). When the temperature exceeds 40 °C, a dual-tiered alarm system is triggered:

- Local alert: A high-decibel buzzer activates for immediate on-site attention.
- Remote alert: Thermal images, temperature data, and timestamps are sent via Telegram API [64,65].

The system also logs temperature trends for predictive maintenance, helping detect early signs of friction or ferromagnetic accumulation and reducing undesired downtime.

#### 4.2.3. Material Edge Detection

**Figure 18:**
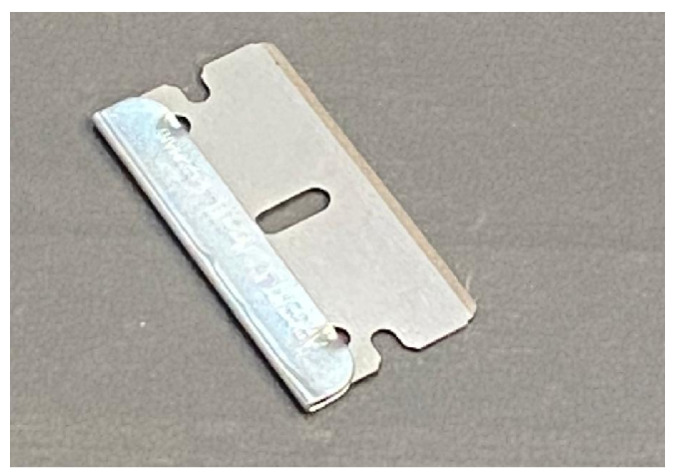
Sharp-edged object on the conveyor belt.

Sharp-edged materials may enter the BECS due to improper sorting or insufficient milling. When ejected during high-speed separation, these fragments risk tearing the conveyor belt and injuring nearby workers. Additionally, they disrupt the generation of stable eddy currents, reducing separation accuracy. Figure 18 illustrates an example of a sharp object present on the conveyor belt.

To address this, the lightweight YOLOv11n-seg model [66] is used to classify particles as either “smooth” or “sharp” based on shape. A high-resolution monochrome line-scan camera is mounted above the belt to capture grayscale images. Smooth particles support stable magnetic interaction, while sharp fragments are flagged as hazardous. This classification helps enhance both safety and separation performance.

#### 4.2.4. Custom Dataset Preparation

In total, we had access to approximately 300 kg of pre-processed recycled materials, ready to enter the eddy current separator stage. As described in the Background section (Section 1.1), these materials typically undergo preliminary treatments such as milling and screening to remove oversized or sharp-edged fragments before reaching the separation stage. Since our samples had already passed through these processes, no raw or unprocessed sharp materials were available for training purposes. Consequently, we had to manually create sharp objects by cutting pieces from aluminum cans to generate training data for the model.

**Figure 19:**
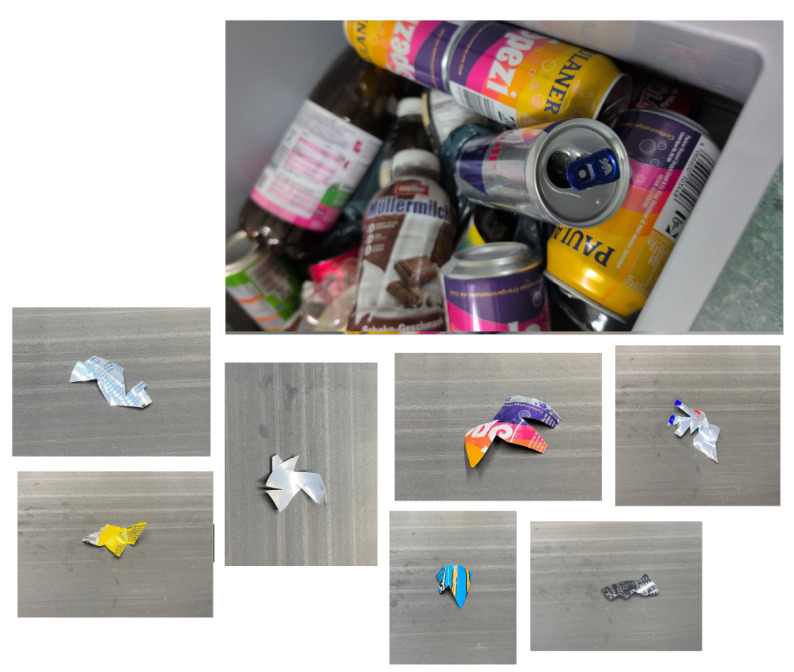
A close-up view of recyclable waste consisting of aluminum cans bottles.

To train a machine learning model capable of distinguishing between sharp-edged and smooth materials, a dedicated dataset was meticulously created using real-world industrial samples. Over one thousand sharp fragments were manually crafted from recycled aluminum cans Figure 19 using cutting tools, mechanical presses, and shaping devices. These objects were intentionally designed with irregular, jagged edges to simulate hazardous elements commonly found in industrial separation systems, which could damage conveyor belts or compromise the quality of separation. In parallel, smooth materials such as rounded pieces of aluminum, copper, and plastic were collected from the output stream of an eddy current separator.

For the imaging phase, at the early stages of the project, several camera types, including ELP 8 Megapixel Webcam with HD 5-50 mm, Raspberry Pi Camera, and GoPro Hero 12, were tested for capturing images of materials moving on the conveyor belt. However, initial experiments revealed that these cameras failed to deliver precise and detailed images at higher belt speeds 6. Notably, in high-speed conditions, the images often lacked edge detail due to motion blur, making it difficult to annotate and train the model with sufficient accuracy.

In contrast, the line scan camera, which acquires images line by line in sync with the conveyor movement, proved to be far more effective. Unlike area scan cameras that capture full frames at once, line scan technology reconstructs a complete image by sequentially capturing the narrow slices aligned with the object’s motion. This method ensures that, even at high speeds, the resulting images remain sharp and rich in edge detail—an essential requirement for our model, which classifies materials based on their edge sharpness.

**Figure 20:**
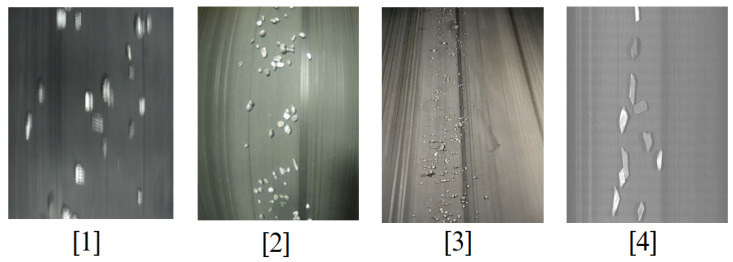
Images of materials moving on a conveyor belt at 6 meters per minute, captured by four different camera types. From left to right: (1) ELP camera, (2) Raspberry Pi camera, (3) GoPro camera, and (4) line scan camera. Only the fourth image offers sufficient clarity and edge detail to enable accurate annotation and effective machine learning training.

As demonstrated in Figure 20, all four images were captured at a constant belt speed of 6. From left to right, they correspond to ELP, Raspberry Pi, GoPro, and Line Scan cameras, respectively. Only the fourth image (line scan), presents the necessary clarity and resolution to reliably detect object contours and enable precise annotation for model training.

Considering the industrial-grade expectations of this project—not just a lab-scale test—the Line Scan camera was the only feasible option capable of delivering high-quality images under real-world conditions. Moreover, between line scan cameras and area scan cameras, as detailed by Basler, line scan cameras capture images one line at a time by synchronizing with the motion of objects, making them especially suitable for continuous processes, such as conveyor belts. In contrast, area scan cameras capture full 2D frames in a single exposure, which can result in motion blur or incomplete edge capture at high speeds. This line-by-line acquisition offers superior resolution and stability when inspecting fast-moving or narrow-field materials, such as those encountered in industrial sorting or recycling systems [67,68].

The monochrome line scan camera was mounted directly above the conveyor belt at a 90-degree angle to ensure the precise top–down image capture of the materials. To provide uniform illumination and minimize shadows or unwanted reflections, two linear light sources were positioned at 45-degree angles on either side of the camera, targeting the conveyor surface. This lighting configuration effectively enhanced the visibility of edges, contours, and shape differences among materials. Throughout the experiments, the conveyor speed was adjusted multiple times to evaluate its impact on image quality. After several tests, it was found that, even at relatively high speeds (above unit 6), the camera was able to maintain acceptable image sharpness as long as frame acquisition was synchronized. This finding confirms the system’s capability to operate at higher speeds without compromising the quality of shape-based analysis in practical scenarios.

Manual annotation was performed using the Roboflow [69] platform. Each object was labeled using polygonal segmentation to trace the outlines of the materials precisely.

**Figure 21:**
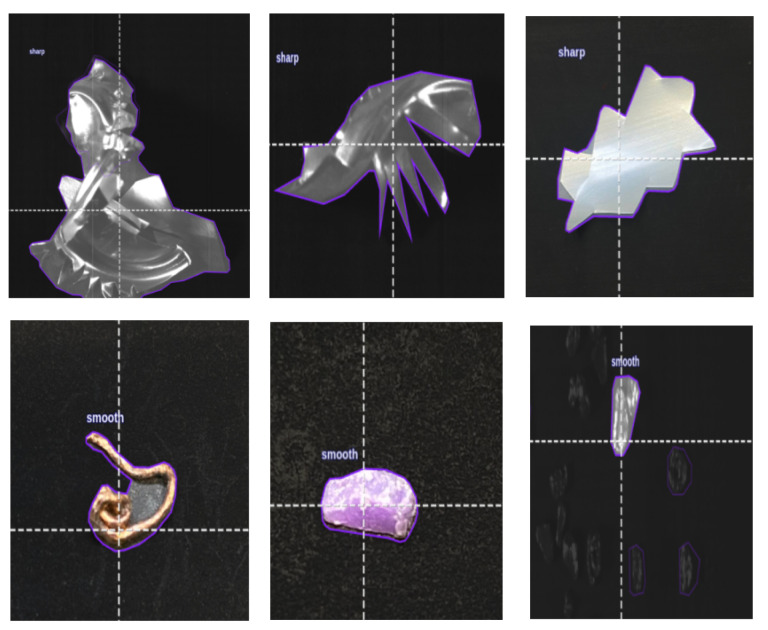
Roboflow-labeled samples: sharp (**top**) and smooth (**bottom**) materials.

In total, 2591 annotated images were produced, comprising 2348 sharp and 3888 smooth objects. As shown in Figure 21, several sharp samples are annotated with purple outlines during the training phase.

Special care was taken to mark geometrically complex and sharp-edged contours, enhancing the model’s ability to learn based on shape attributes rather than color or texture alone. The annotated dataset was then randomly split into training (70%), validation (20%), and testing (10%) subsets, with class distribution carefully balanced across all phases to avoid training bias. Preprocessing procedures were applied, including the removal of incorrectly labeled instances, the elimination of image noise, the validation of overlapping labels, and the correction of image alignment. This finalized and curated dataset served as a reliable input for training the YOLOv11-based edge classification model used in our intelligent material detection system for recycling applications.

In contrast, for smooth or rounded-edge materials, no manual generation was necessary, as a sufficient quantity of such objects already existed in the collected dataset. Therefore, only imaging, annotation, and edge feature extraction were performed for those instances.

#### 4.2.5. YOLOv11-Based Edge Detection

YOLOv11 is a fast, one-stage object detection model ideal for real-time tasks. YOLOv11n-seg, its lightweight version used in this project, supports both detection and segmentation on low-resource devices, offering a strong balance between speed and accuracy [70,71,72].

YOLOv11 was selected due to its high accuracy in detecting small and complex objects, combined with fast inference, making it suitable for deployment on our GPU-equipped laptop. Unlike heavier models like YOLOv5-L or Faster R-CNN, YOLOv11 provides an optimal trade-off between speed and accuracy, making it appropriate for high-throughput industrial conveyor systems [73,74,75].

Moreover, its superior accuracy in detecting complex objects makes it ideal for real-time applications on GPU-enabled edge devices. Prior studies, such as [76], have shown that YOLO architectures offer a favorable balance between performance and speed, especially in high-throughput industrial environments.

In newer versions of YOLO (such as YOLOv11), improvements in the FPN and PANet architectures have enhanced the detection accuracy of small objects with fine details, including recyclable particles measuring 1 to 4 mm [77].

The detection process involves several key steps:

1. The input image is passed through a series of convolutional layers to extract spatial features.
2. The image is divided into a grid; each grid cell predicts bounding boxes and class labels.
3. The model outputs the predicted location (*x*, *y*, *w*, *h*), class label (e.g., “sharp” or “smooth”), and a confidence score.
4. In the segmentation version, a pixel-wise mask is also generated for each detected object.

Each output includes the following elements:

1. Bounding box coordinates.
2. Class label (e.g., sharp or smooth).
3. Segmentation mask.
4. Confidence score (e.g., 0.93).

Training the model involves a composite loss function that captures different aspects of detection and segmentation performance:

TotalLoss = BoxLoss + ClassLoss + DFLLoss + SegmentationLoss [78]

where

1. BoxLoss: Penalizes inaccuracies in bounding box predictions.
2. ClassLoss: Measures classification error using cross-entropy.
3. DFLLoss: Distribution Focal Loss for better localization precision.
4. SegmentationLoss: Assesses pixel-level segmentation accuracy.

### 4.3. Smart Separation

**Figure 22:**
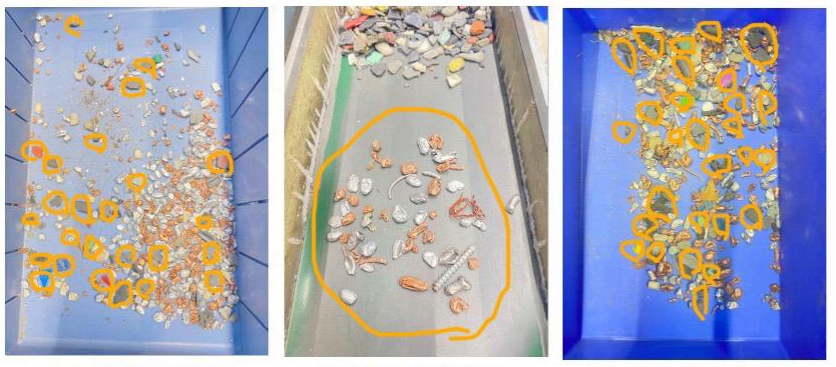
Examples of separation errors in a BECS system: (**left** and **right**) plastics incorrectly sorted into the metal bin; (**center**) metals mistakenly deposited in the plastic stream.

The efficient separation of non-ferrous metals and plastics is crucial for reducing energy consumption and facilitating reprocessing. This work optimizes BECS performance comprising a conveyor, feeder, and magnetic drum by replacing manual, experience-based adjustments with more intelligent calibration. As shown in Figure 22, poor settings can cause frequent misclassifications.

We present a data-driven approach to optimize BECS settings. Using 82 experimental tests with varying speeds, vibration levels, and drum angles, machine learning identified configurations that reduced sorting errors and energy use. Compared to manual tuning, this method improves accuracy, efficiency, and sustainability in industrial recycling.

#### 4.3.1. Constructing a Reliable Dataset for Machine Learning-Based BECS Optimization

**Figure 23:**
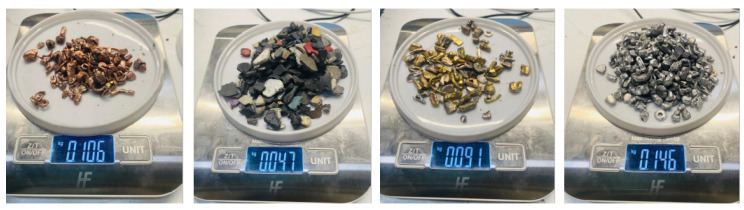
Weight measurements of misclassified materials collected from the output bins: (**left** to **right**) copper in plastic bin, plastic in metal bin, brass in plastic bin, and aluminum in plastic bin.

The BECS separates non-ferrous metals, such as aluminum, copper, and brass, from mixed waste [79]. To optimize performance, this study experimentally investigates the effects of belt speed, vibration intensity, and drum angle across 81 controlled trials, with the drum speed maintained at 67.40 Hz. After each trial, misclassified materials are weighed to assess separation accuracy. Figure 23 illustrates the weight measurements of these collected misclassifications.

The types of errors recorded include

- Copper particles mistakenly sorted into the plastic stream.
- Plastic particles incorrectly deposited in the metal bin.
- Brass fragments misclassified as plastic.
- Aluminum components misdirected into the plastic fraction.

Key parameters like belt speed, drum speed, vibration, and angle affect separation quality. A multi-output regression model is used to predict optimal settings to boost accuracy and cut costs. This section outlines data preparation, model training, and the optimization process.

#### 4.3.2. Dataset and Data Preparation

The dataset used in this analysis is collected from a functioning industrial BECS unit and includes both the operational settings and corresponding separation error metrics. The data is organized into two main categories:

Input Features (X):
  - Vibration speed (low, medium, high);
  - Conveyor belt speed (Hz);
  - Separation errors for aluminum, copper, brass, and plastic (grams);
  - Drum angle (degrees).

Output Variables (y):
  - Optimized vibration speed;
  - Optimized conveyor belt speed;
  - Optimized drum angle.

During pre-processing, missing data is addressed, and the MinMaxScaler is applied to normalize all input features, ensuring that each variable is rescaled to a standard range, thus improving the model accuracy. The dataset is split into 80% for training and 20% for testing to evaluate generalization.

#### 4.3.3. Machine Learning-Based Approach

The optimization task is formulated as a multi-output regression problem [80], implemented using the Scikit-learn library [81]. This type of model is particularly suited for predicting multiple interrelated output variables simultaneously. For this task, a random forest regressor [82] is selected as the base learner due to its robustness in modeling nonlinear relationships and its strong performance on structured datasets.

The multi-output regression problem is expressed as

(2) $$y_{j}=f_{j}\left(X\right)+\epsilon_{j},\;j=1,2,\ldots ,m$$

where

- $X=(x_{1},x_{2},\ldots ,x_{n})$ is the vector of input features, consisting of
  – $x_{1}$: vibration speed (low, medium, high);
  – $x_{2}$: conveyor belt speed (Hz);
  – $x_{3}$: separation error for aluminum in plastics (grams);
  – $x_{4}$: separation error for copper in plastics (grams);
  – $x_{5}$: separation error for brass in plastics (grams);
  – $x_{6}$: separation error for plastics in metals (grams);
  – $x_{7}$: drum angle (degrees).
- $y_{j}$ represents the predicted optimized operational settings, including
  – $y_{1}$: optimized vibration speed (low, medium, high);
  – $y_{2}$: optimized conveyor belt speed (Hz);
  – $y_{3}$: optimized drum angle (degrees).
- $f_{j}\left(X\right)$ is a nonlinear function approximated by the random forest model, trained to find the optimal settings for the BECS system.
- $\epsilon_{j}$ represents the model error, accounting for the discrepancy between the predicted and actual optimal operational settings.

The selected model is trained on normalized data to learn optimal BECS settings and validated on unseen samples. Its goal is to recommend configurations that reliably improve separation accuracy and efficiency.

### 4.4. Integration of AI and Internet of Things with PLC

Two safety-critical modules are integrated into the BECS system’s PLC: Thermal fire detection and sharp-edge material detection. The thermal module monitors heat near the magnetic drum. It shuts down the system via PLC while sending a Telegram alert if temperatures rise dangerously—preventing fires caused by trapped ferromagnetic materials. The sharp-edge module halts the system when excessive hazardous fragments are detected on the belt, prompting manual inspection.

**Figure 24:**
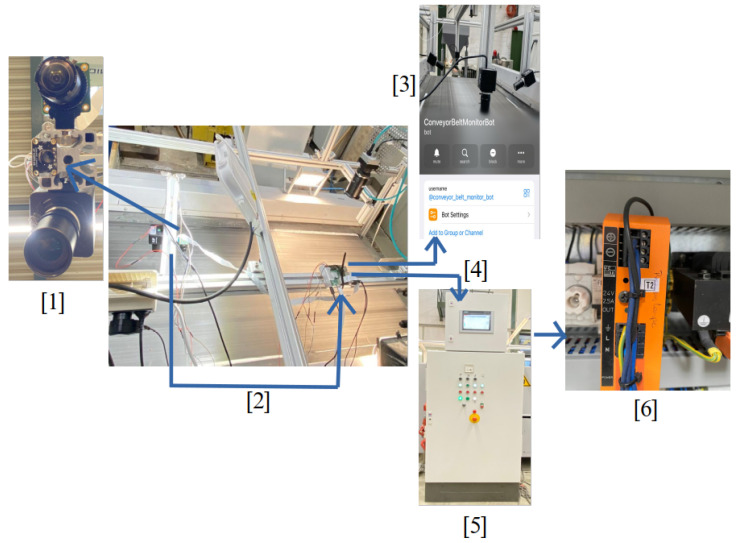
(1) Thermal camera used to monitor temperature above the conveyor belt. (2) Connection of the thermal camera to the Raspberry Pi for image processing. (3) Remote alert sent via Telegram bot for immediate notification. (4) PLC module mounted above the manual control unit and linked to the Raspberry Pi. (5) Manual control panel for operating the BECS system. (6) Power supply for the Raspberry Pi, located inside the PLC cabinet.

These automated responses replace manual intervention in traditional BECS systems, improving safety and efficiency. In contrast, the belt misalignment correction runs independently via lightweight IoT hardware and stepper motors, performing non-critical, local adjustments. Figure 24 shows the full integration of the fire detection system, starting with thermal image capture by a FLIR or MLX90640 camera (mounted near the drum), followed by real-time analysis on a Raspberry Pi using Adafruit and OpenCV. The 24 × 32 temperature matrix is processed to extract the maximum temperature in each frame.

If the detected temperature exceeds a critical threshold (e.g., 40 °C), the Raspberry Pi triggers a three-part emergency response:

1. A thermal image is captured, annotated with the temperature value, and stored.
2. A remote warning is sent via a Telegram bot, including the annotated image, to notify the technician.
3. A shutdown command is issued to the Siemens PLC using the OPC UA protocol.

The shutdown command is transmitted to the PLC via an OPC UA connection, using the Python opcua library.

The PLC node responsible for shutdown is identified:

1. As ns = 2, s = OPC_Daten.Anlage_ausschalten
2. A Boolean value of true is written to this node, deactivating the entire system.

Automatic shutdown is essential when ferromagnetic materials get trapped near the magnetic drum, generating heat through friction. If unchecked, this can damage or ignite the conveyor belt. Prompt shutdown turns off the magnetic field, stops heat buildup, and prevents further damage.

## 5. System Testing and Evaluation

To address the research objectives, the system is evaluated across three primary axes corresponding to the research questions: Accident prevention, improved material separation under high throughput, and the role of material identification in enhancing separation quality.

To prevent unwanted incidents in the conveyor system, a series of intelligent modules were developed and evaluated across three critical aspects: Misalignment correction, fire hazard detection, and sharp particle identification. These measures not only reduce the risk of mechanical damage and classification errors but also play a vital role in protecting worker safety and maintaining workplace integrity.

**Figure 25:**
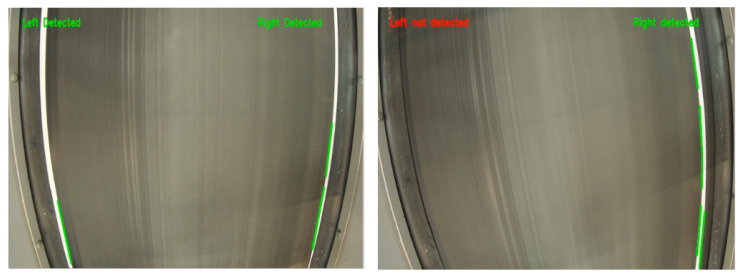
Left image shows edge detection results: Both edges detected while the right image shows that the left edge has not been detected.

First, a camera-based misalignment detection system using OpenCV (Canny + Hough Transform) was implemented. It achieved over 90% detection accuracy under varying lighting and vibration conditions. Upon detecting a deviation, the system activated the stepper motors to realign the belt, completing detection and correction in under 0.08 s. The result has been shown in Figure 25.

**Figure 26:**
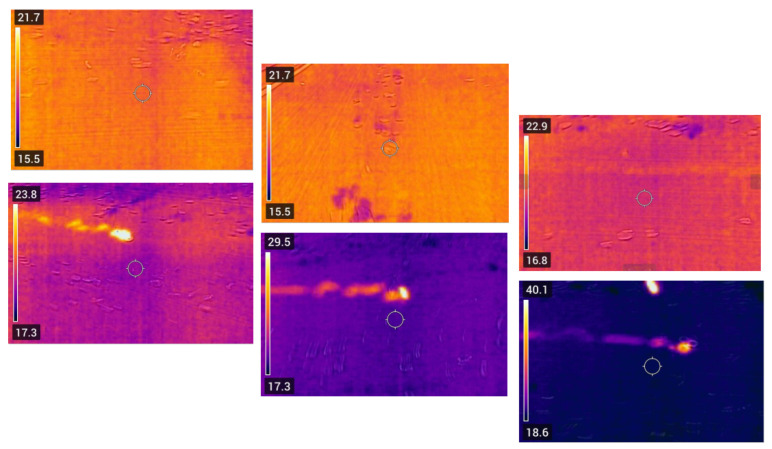
The thermal image sequence captures the presence of an actual iron particle on the conveyor belt, specifically in the section located above the magnetic drum. The resulting friction and magnetic retention in this area caused a sudden increase in temperature.

Second, a thermal monitoring unit using the MLX90640 camera was deployed across five industrial scenarios. The system achieved a 97% success rate in detecting abnormal heat rises, especially in cases where ferromagnetic objects became trapped under the belt near the magnetic drum. Alerts were delivered via buzzer and Telegram, and the system’s average response time ranged from 1.7 to 2.5 s. This rapid detection effectively reduced the risk of overheating and fire escalation. The result has been shown in Figure 26 and Table 1.

**Figure 27:**
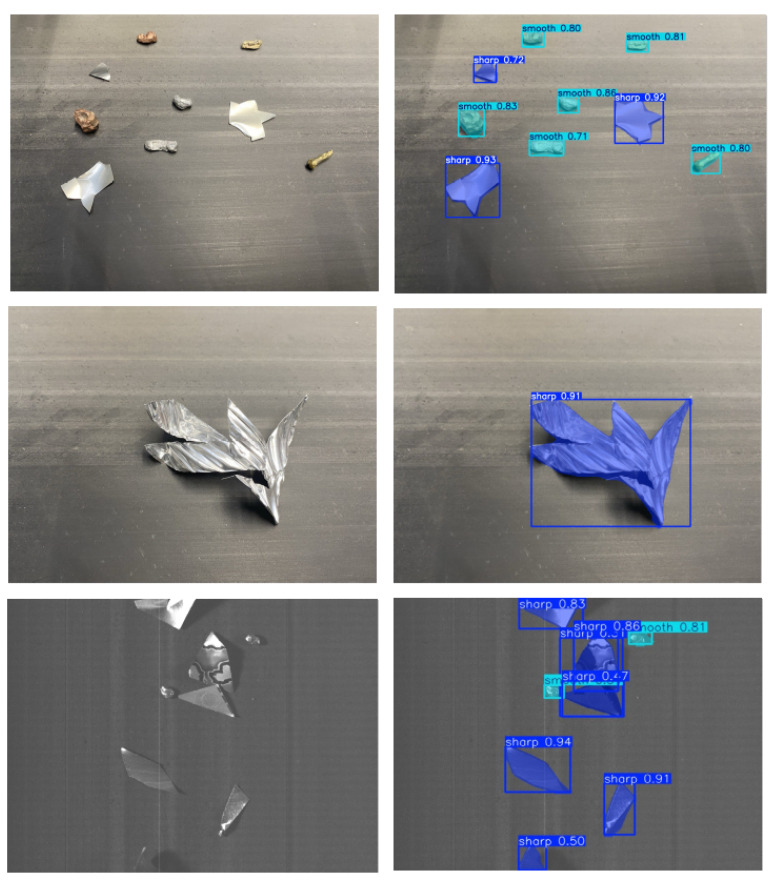
Detection results of mixed sharp and smooth objects using both RGB and mono line-scan cameras, showing the original image on the left and the segmented output on the right.

Finally, a YOLOv11n-seg model was trained to classify particle shapes (sharp-edged vs. smooth) using line-scan camera images. This module helps prevent belt tearing and damage to the magnetic drum caused by sharp fragments. Additionally, sharp-edged particles can disrupt the material flow and reduce separation quality if not correctly identified. By detecting such particles in advance, the system ensures more stable separation and reduces operational disruptions. Moreover, it enhances operator safety by lowering the risk of mechanical failure or material ejection in high-speed environments. The results are shown in Figure 27.

To improve separation accuracy without reducing throughput, a machine learning model (MultiOutputRegressor + Random Forest) was trained on 82 real test runs involving 500 g samples of various materials. The model learned from misclassification patterns and predicted optimal settings for vibration, conveyor, and drum. Applying these recommendations led to notable improvements:

- Aluminum: 96.2% accuracy (19 g error)
- Copper: 98.2% accuracy (9 g error)
- Brass: 97.0% accuracy (15 g error)
- Plastic: 94.8% accuracy (26 g error)

**Figure 28:**
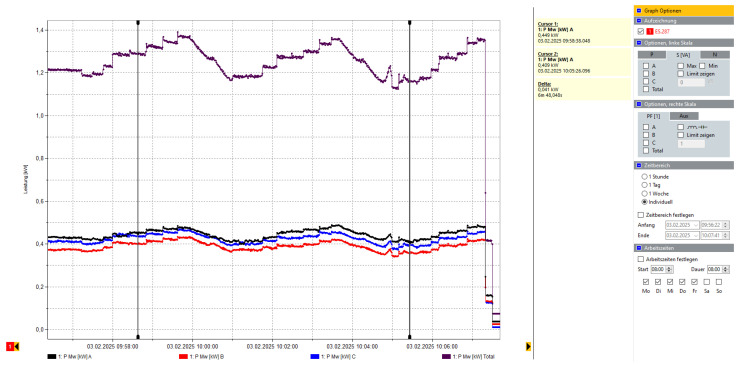
Energy consumption before machine learning optimization.

**Figure 29:**
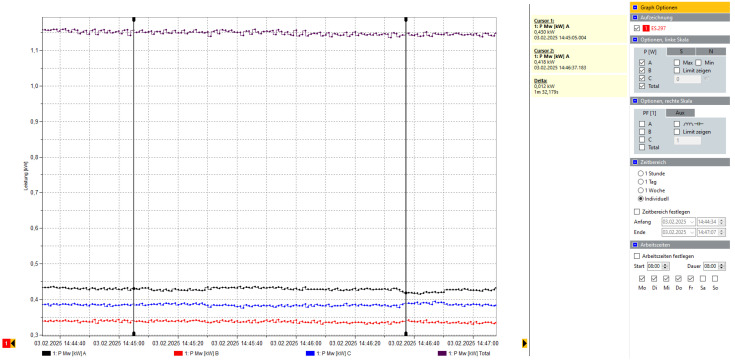
Energy consumption after machine learning optimization.

Power usage analysis revealed a 15–18% reduction in energy consumption after model-based parameter tuning, highlighting both operational and environmental benefits. The results are shown in Figure 28 and Figure 29.

**Figure 30:**
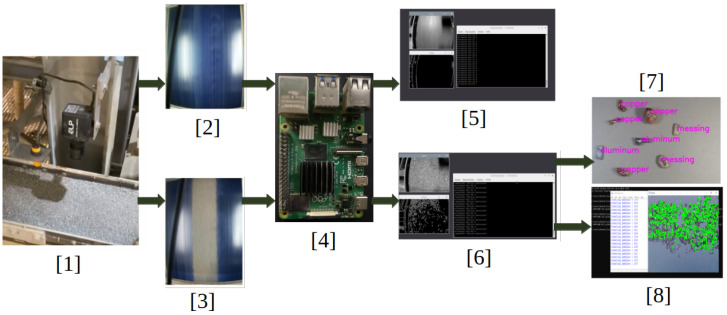
(1) Mounted motion sensor and an ELP camera above the vibration feed; (2) Empty belt; (3) Belt with material moving; (4) The processor; (5) Empty belt results absence of material; (6) Belt with material results presence of material; (7) Detected type of material; (8) Counted the amount of material.

Two methods of recognition modules were developed. A color-based system (using K-means and a decision tree on a Raspberry Pi) achieved 100% accuracy for aluminum and 97% copper and over 93% accuracy for other types, which the overview of setup and result in depicted in Figure 30 and for high-speed scenarios, a YOLOv11n-seg deep learning model was trained on a custom dataset. The model delivered outstanding metrics:

- mAP@50: 0.994, mask recall: 0.979
- Class-wise mAP: aluminum 0.993, copper 0.991, plastic 0.995
- Inference speed: ∼6 ms per image

**Figure 31:**
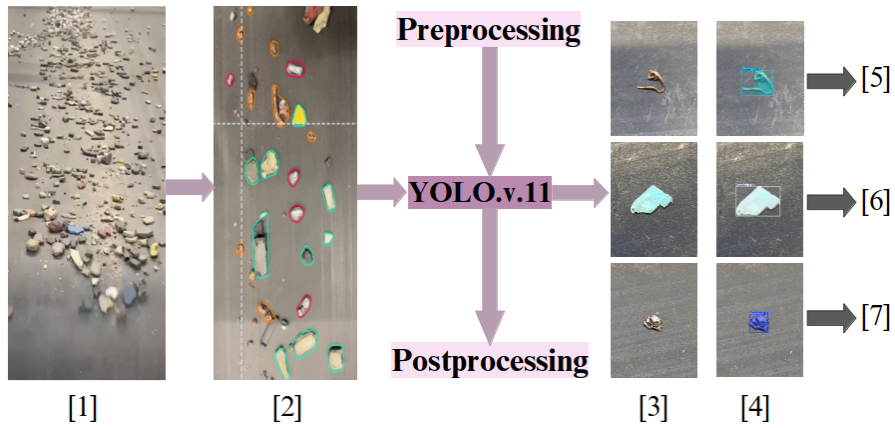
(1) Original image of material; (2) Annotated image of material; (3) Original images of samples being test; (4) Detected images of samples being test; (5) Detected copper; (6) Detected plastics; (7) Detected aluminum.

The result are presented in Figure 31.

### 5.1. Discussion

**Figure 32:**
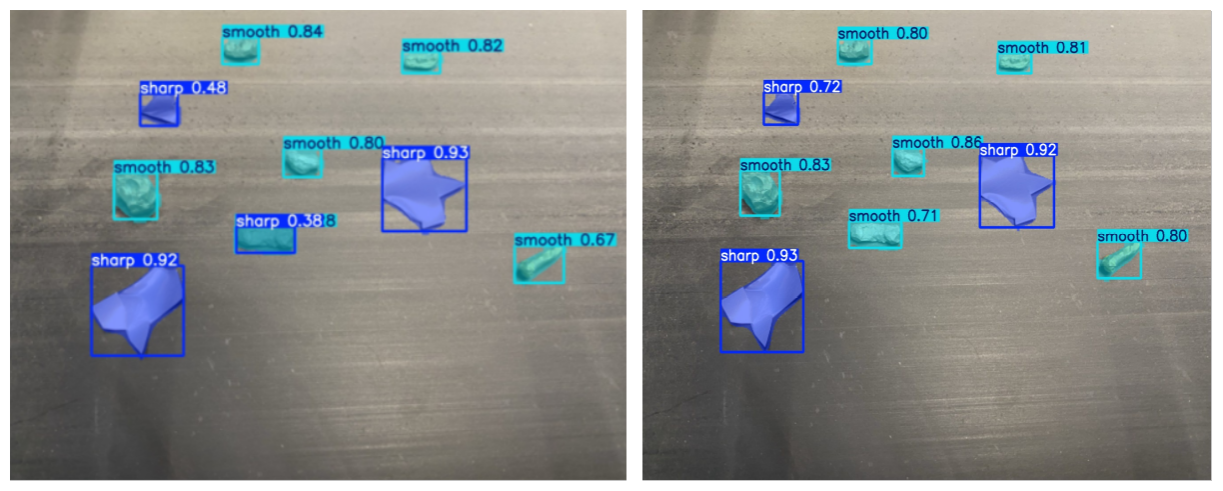
Comparison of YOLOv8 (**left**) and YOLOv11 (**right**) models in detecting and classifying objects with sharp and smooth edges on a conveyor belt. The YOLOv11 model demonstrates higher confidence scores and more accurate localization, particularly for sharp-edged objects. It successfully detects smaller and less visible particles that YOLOv8 either misclassifies or misses. Overall, YOLOv11 exhibits improved performance with fewer false negatives and better shape differentiation, making it more suitable for real-time industrial applications in material shape recognition.

The side-by-side comparison of Figure 32 illustrates the output performance of two object detection models—YOLOv8 and YOLOv11—when applied to detect material shapes, particularly distinguishing between “sharp” and “smooth” objects on a conveyor belt. The analysis clearly shows that YOLOv11 outperforms YOLOv8 in multiple dimensions, including confidence levels, detection coverage, and classification stability.

**Figure 33:**
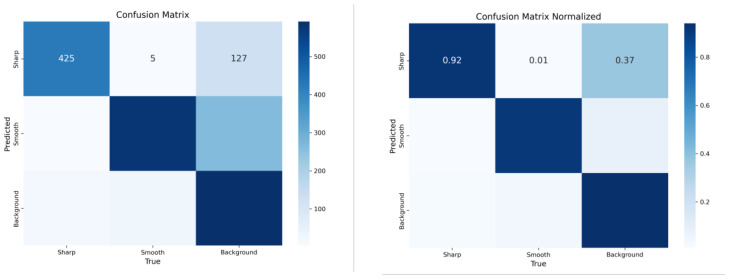
Confusion matrix (**left**) and normalized confusion matrix (**right**) of the YOLOv11 model for classifying three categories: sharp, smooth, and background. The left matrix displays the absolute number of predictions, while the right matrix shows normalized values. Although the model performs well overall, some misclassifications—particularly between sharp objects and background—highlight areas for potential improvement in preprocessing or model tuning.

In terms of detection confidence, YOLOv11 consistently produces higher confidence scores across the board. For instance, although both models detect the centrally located sharp object with a confidence of 0.93, the sharp item at the top–left is detected with a significantly better score by YOLOv11 (0.72) compared to YOLOv8 (0.48). Furthermore, YOLOv11 captures all instances identified by YOLOv8 and even adds more accurate bounding boxes with fewer misclassifications and weak predictions. Especially in edge areas of the frame, YOLOv11 displays greater stability in labeling “smooth” materials, which is crucial in real-time industrial applications where objects might appear partially cropped or blurred. To validate the model’s performance quantitatively, a comprehensive analysis of the confusion matrix which has been shown in Figure 33 was conducted.

Out of 557 “Sharp” samples in the test dataset, 425 were correctly predicted, while only five were misclassified as “Smooth”, and 127 were mistakenly labeled as “background”. The normalized matrix confirms that the YOLOv11 model achieved an accuracy of approximately 92% in correctly identifying sharp-edged objects, illustrating the model’s high precision and its effectiveness in minimizing false positives. This is particularly vital in industrial quality control scenarios, where the incorrect detection of sharp objects may lead to mechanical hazards or misrouting.

Training and validation metrics over 300 epochs support the model’s stability and reliability. All loss components—box, segmentation, classification, and distribution focal loss—show a smooth and consistent downward trend without signs of overfitting. Performance metrics further highlight the model’s strength, with precision reaching approximately 0.83, recall around 0.86, and mAP@0.5 exceeding 0.9, while mAP@0.5–0.95 is maintained near 0.65. These results reflect robust detection capabilities even for fine-grained, small-scale materials, validating the model’s generalization to unseen cases.

Finally, the dataset’s distribution analysis reveals a well-balanced class representation between “Sharp” and “Smooth” materials, with each comprising between 1600 and 1900 labeled samples. Bounding box positions are evenly distributed across the frame, and the variety in object sizes and locations ensures that the dataset captures realistic conditions on the conveyor belt. This diversity contributes positively to the model’s adaptability and robustness in real-world deployment scenarios.

The full composite image comprises 16 plots that display the training and validation process of a YOLO-based model (likely YOLOv8 or YOLOv11) across 300 epochs. These plots track the evolution of losses and evaluation metrics such as precision, recall, and mAP for different datasets or classes.

**Part 1—Training Loss Curves:**

**Figure 34:**
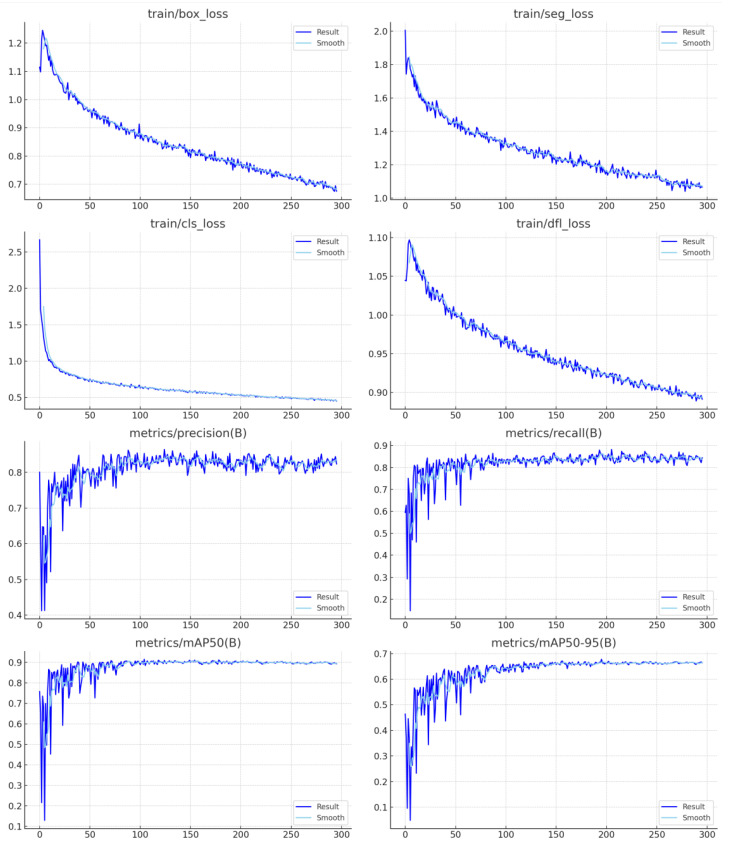
Summary of the YOLOv11 model’s training performance for detecting particles with sharp and smooth edges on a conveyor belt. The plots display the decreasing trends of key loss components.

**Figure 35:**
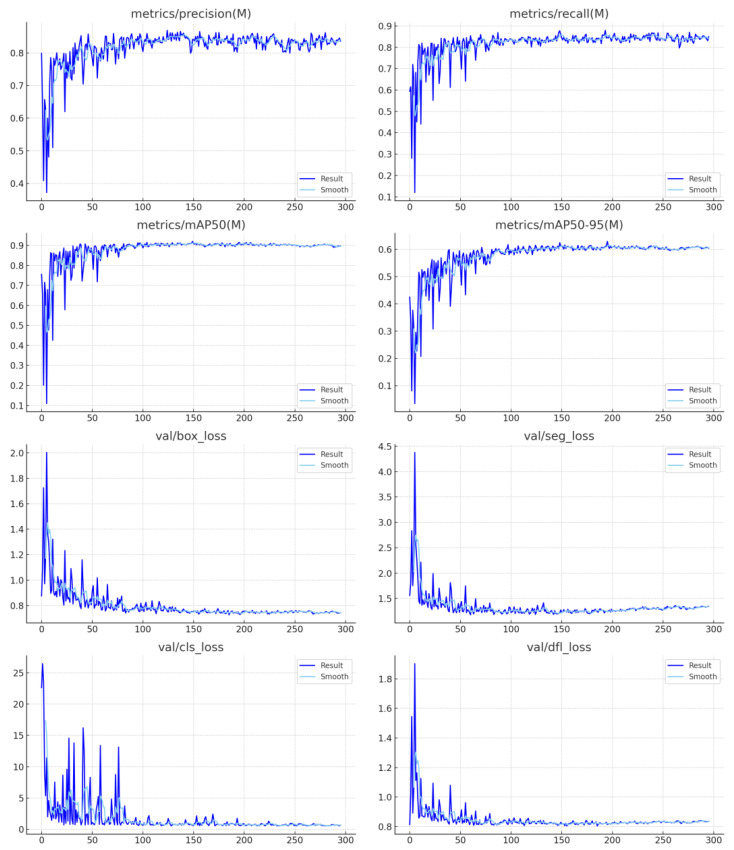
Continuation of the performance summary, focusing on validation results. The plots illustrate the convergence of validation loss components and the stabilization of evaluation metrics (precision, recall, mAP@50, and mAP@50–95).

In Figure 34 and Figure 35 show how various loss components evolve during training:

- train/box_loss shows a smooth and steady decline from around 1.2 to below 0.7, reflecting better localization accuracy of bounding boxes as training progresses.
- train/seg_loss also steadily decreases, indicating the model’s improved ability to perform precise segmentation of objects.
- train/cls_loss drops sharply in the early epochs and stabilizes, which indicates the model quickly learns to classify objects correctly.
- train/dfl_loss (Distribution focal loss) steadily decreases, improving localization precision in predicted object positions.

**Part 2—Evaluation Metrics for Class B and Class M:**

The middle two rows track precision, recall, and mAP values for both classes:

- metrics/precision(B) and metrics/recall(B), as well as their counterparts for class M, show consistent improvement and stabilize around 0.8 to 0.9, indicating strong classification performance for both datasets.
- metrics/mAP50(B) and metrics/mAP50(M) reach close to 0.9, indicating high detection accuracy at a relaxed IoU threshold.
- metrics/mAP50-95(B) and metrics/mAP50-95(M) stabilize around 0.6, which is very good for strict IoU thresholds from 0.5 to 0.95.

These trends confirm that the model is generalizing well and not overfitting.

**Part 3—Validation Loss Curves:**

The bottom four plots show how the loss values evolve on the validation dataset:

- val/box_loss, val/seg_loss, val/cls_loss, and val/dfl_loss show significant fluctuation in early epochs but gradually settle, a typical behavior when adapting to unseen data.
- The initial spikes, especially in val/cls_loss, suggest either a higher variability in validation data or class imbalance, but the trend improves over time.

### 5.2. Limitations

**Figure 36:**
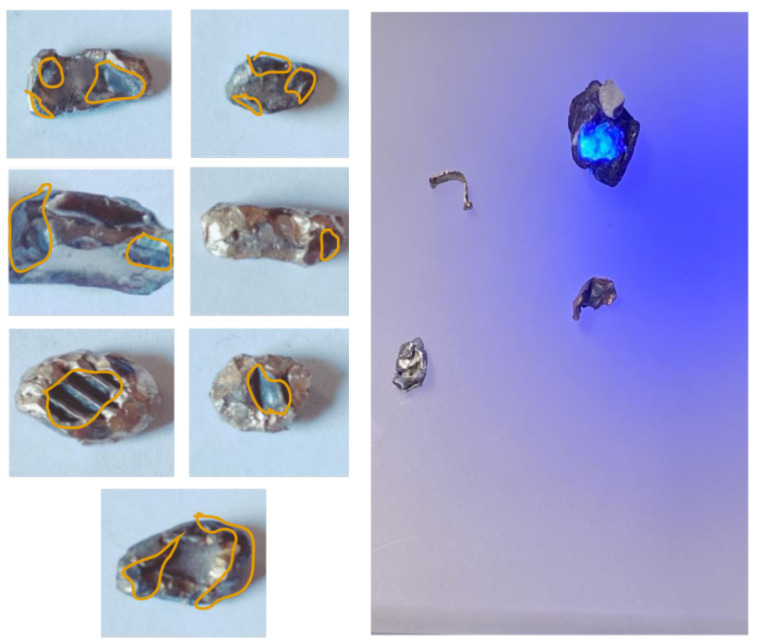
Examples of composite and coated materials with heterogeneous surface textures and layered structures. The left panel highlights distinct regions within each sample using orange contours, indicating mixed textures or coatings. These variations pose challenges for straightforward classification. The right panel shows the same samples under UV lighting to reveal fluorescence or reflective differences caused by surface coatings. These materials were used in the PiVisionSort project to evaluate classification algorithms based on K-Means and decision tree models.

Despite the promising results and comprehensive integration achieved in the Twin-AI BECS system, several limitations remain that should be addressed in future research:

- While the YOLOv11 and YOLOv11n-seg models demonstrated high accuracy in material and shape classification, deploying them on low-powered edge devices, such as the Raspberry Pi or Jetson Nano, still presents computational and memory limitations. Near-real-time inference under harsh industrial conditions remains partially dependent on offloading to more powerful local servers or optimizing the model architecture. To explore lighter alternatives, YOLOv5 and YOLOv8 models were also tested. YOLOv5 showed significantly lower detection performance and is not recommended for industrial or real-time scenarios. However, YOLOv8 provided reasonably good results with moderate accuracy and better efficiency, making it suitable for users with limited computational resources or tighter budgets. All training datasets and source codes for YOLOv8 are available in our public GitHub repository. For applications where lower cost outweighs maximum accuracy, YOLOv8 is a practical choice. Conversely, for projects requiring high detection precision and near real-time performance, applying the YOLOv11 model remains the most effective option.
- The current classification modules primarily focus on four material types: aluminum, copper, brass, and plastics. Although sufficient for many recycling applications, the system’s performance on composite, coated, or contaminated materials has not been directly evaluated on the conveyor belt. However, this issue was addressed in our previous project, PiVisionSort, which was implemented on a vibration feeder system, shown in Figure 36. In that project, a color-based classification approach was combined with a K-means clustering algorithm to detect material types initially. For composite or coated samples with mixed or ambiguous color patterns, a secondary algorithm—decision tree—was introduced. The decision tree received the K-means output and applied a threshold-based rule (e.g., if 80% of the pixels were classified as orange, the material was categorized as copper). This hybrid method significantly improved classification accuracy to over 97%. Tested composite and coated material samples are illustrated in Figure 31. The entire experiment was performed on the vibration feeder setup and published in the PiVisionSort paper [28]. In future work, we plan to investigate this issue further using hyperspectral imaging technology to assess whether spectral analysis can yield more accurate and robust classification results for composite and coated materials.
- During all experimental scenarios, the magnetic drum speed was held constant to ensure mechanical safety. While this approach simplifies optimization, it restricts the ability of the model to explore dynamic drum speed adjustments, which may provide additional gains in separation accuracy under varying input conditions.
- The shape detection model uses a monochrome line-scan camera to distinguish between sharp and smooth materials. While effective for edge recognition, the lack of color limits the system’s ability to perform material identification or distinguish similarities between the materials.

**Figure 37:**
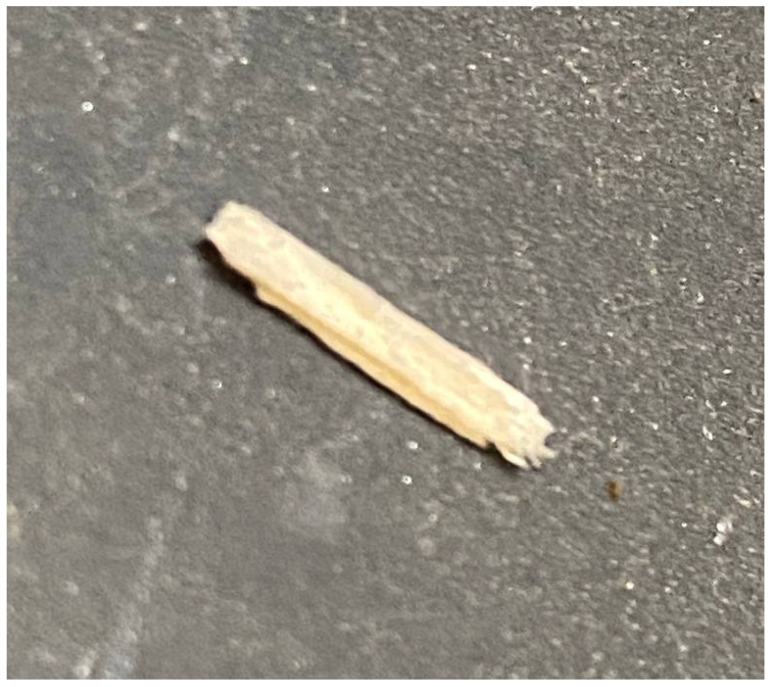
Original image of a piece of wood that was mistakenly included among the recyclable materials.

**Figure 38:**
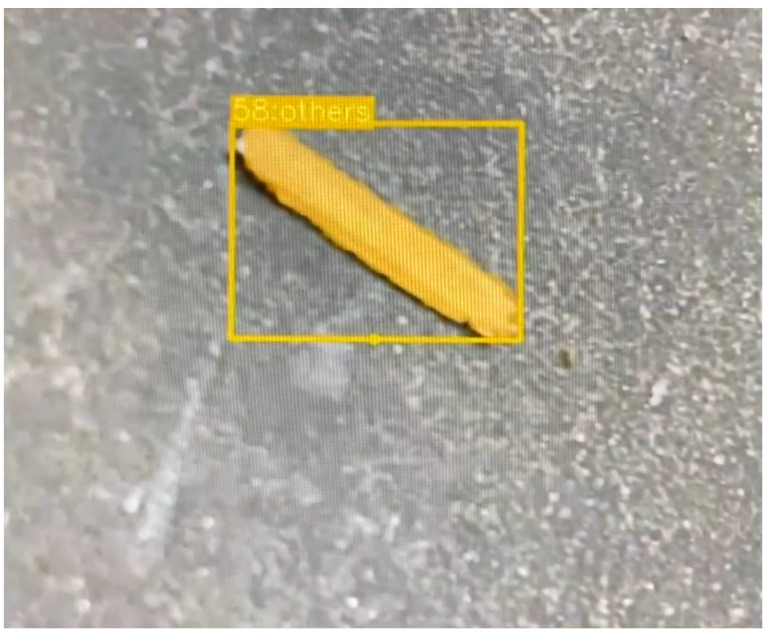
The detected image of wood has been classified under the "Others" category.

The current research integrated a general category labeled “others”. This category is intended to capture any material not classified as aluminum, copper, or plastic—such as wood, glass, or unknown particles—that may accidentally appear on the conveyor belt. As illustrated in Figure 37, a wooden particle mistakenly present in the input stream was successfully identified under the “others” class in Figure 38. This approach not only prevents the misclassification of foreign materials but also enables the system to detect anomalies, paving the way for potential integration in broader recycling or sorting workflows. By allowing such flexibility, the model can be retrained or expanded with new classes depending on the nature of the waste stream, facilitating scalability to diverse industrial recycling environments.

## 6. Conclusions and Future Work

This work presents Twin-AI BECS, a fully integrated intelligent system for optimizing barrier eddy current separators (BECSs) in real-world industrial environments. By combining machine learning, machine vision, IoT-based monitoring, and a digital twin, the system successfully addresses longstanding challenges, including manual tuning, throughput optimization, poor separation accuracy, and conveyor belt misalignment—issues often overlooked in prior studies. Unlike earlier works on isolated subsystems, our approach implements synchronized optimization of the conveyor, drum’s angle, and vibration feed speeds using real industrial data across 82 scenarios, significantly improving separation quality and operational efficiency.

Key contributions include the first real-world deployment of a digital twin in BECS systems, a hybrid vision module that detects particle shape (sharp vs. round), a thermal monitoring system capable of triggering emergency shutdown via PLC, and an intelligent classification module based on YOLOv11 and decision trees. Each module is designed for high-performance and cost-effective deployment using Raspberry Pi and industrial cameras.

Together, this forms a responsive, modular architecture that bridges the gap between theoretical modeling and an industrial application. The proposed system ensures operational stability and predictive maintenance, setting a new benchmark for safety and sustainability in electronic waste recycling.

While the current model (YOLO.v11) effectively distinguishes between key materials such as aluminum, copper, brass, and plastics, future iterations may incorporate advanced hyperspectral or FTIR imaging modules to more precisely classify a wider range of composite, coated, or contaminated materials. Although the YOLO.v11 and YOLOv11n-seg models have demonstrated strong performance, deploying them on constrained edge hardware, such as the Jetson Nano or Coral TPU, requires further comparison and quantization techniques, e.g., pruning and knowledge distillation, to ensure near real-time inference with reduced energy consumption.

The current shape detection system utilizes a monochrome line-scan camera, which limits material identification due to the lack of color data. Future versions could adopt RGB or multi-color line-scan cameras to enable the simultaneous classification of shape and material type, improving overall accuracy and reducing the need for separate detection modules.

Although blockchain technology offers significant potential for traceability and transparency in large-scale recycling networks, it did not apply to the current scope of our project. Our focus was on real-time material classification using machine vision and AI to enhance separation efficiency at the conveyor level. Since our system operates as a local intelligent detection module, the need for distributed, immutable data logging across stakeholders was outside the intended functionality of our system. However, in future industrial deployments where end-to-end traceability of material flow is required—from source to final sorting—blockchain integration could offer added value. 

## Figures and Tables

**Table 1:** Temperature and response time in different scenarios.

ID	Scenario	Average Temp (°C)	Max Temp (°C)	Response Time (s)
0	No Iron Present	19.0	21.7	1.8
1	Iron Present (Low Speed)	29.5	42.0	2.1
2	Iron Present (High Speed)	40.1	45.3	2.3
3	Iron and Dust Present	38.5	43.8	2.5
4	Mixed Materials (No Iron)	20.5	22.0	1.7

## Data Availability

The dataset supporting the findings of this study are publicly available under the name *SoRec Project* on the Göttingen Research Online (GRO.data) platform. The dataset can be accessed at https://data.goettingen-research-online.de/dataverse/BECS-Smart-Separation-Data (accessed on 29 June 2025). Additionally, a limited portion of the dataset, along with the source code used in this study, is available on GitHub at https://github.com/ETCE-LAB/SoRec-General (accessed on 29 June 2025).

## References

[1] Forti V., Baldé C.P., Kuehr R., Bel G. (2020). The Global E-Waste Monitor 2020.

[2] Cucchiella F., D’Adamo I., Koh S., Rosa P. (2015). Recycling of WEEEs: An economic assessment of present and future e-waste streams. Renew. Sustain. Energy Rev..

[3] Ramanayaka S., Santhirasekaram K., Vithanage M. (2019). Urban mining of E-waste: Treasure hunting for precious nanometals. Handbook of Electronic Waste Management.

[4] Aniley A.A., Simegn A.A. (2023). A Comprehensive Review on Challenges and Opportunities of e-waste Management Practices in Ethiopia. ASRIC J. Soc. Sci. Humanit..

[5] Luther L. (2010). Managing Electronic Waste: Issues with Exporting E-Waste.

[6] Smith Y.R., Nagel J.R., Rajamani R.K. (2019). Eddy current separation for recovery of non-ferrous metallic particles: A comprehensive review. Miner. Eng..

[7] Rem P., Leest P., Van den Akker A. (1997). A model for eddy current separation. Int. J. Miner. Process..

[8] Jujun R., Yiming Q., Zhenming X. (2014). Environment-friendly technology for recovering nonferrous metals from e-waste: Eddy current separation. Resour. Conserv. Recycl..

[9] Aschenbrenner D., Gros J., Fangerow N., Werner T., Colloseus C., Taha I. (2023). Recyclebot–using robots for sustainable plastic recycling. Procedia CIRP.

[10] Lubongo C., Bin Daej M.A., Alexandridis P. (2024). Recent developments in technology for sorting plastic for recycling: The emergence of artificial intelligence and the rise of the robots. Recycling.

[11] Wilts H., Garcia B.R., Garlito R.G., Gómez L.S., Prieto E.G. (2021). Artificial intelligence in the sorting of municipal waste as an enabler of the circular economy. Resources.

[12] Cheng T., Kojima D., Hu H., Onoda H., Pandyaswargo A.H. (2024). Optimizing Waste Sorting for Sustainability: An AI-Powered Robotic Solution for Beverage Container Recycling. Sustainability.

[13] Lakhouit A. (2025). Revolutionizing Urban Solid Waste Management with AI and IoT: A review of smart solutions for waste collection, sorting, and recycling. Results Eng..

[14] Bin C., Yi Y., Zhicheng S., Qiang W., Abdelkader A., Kamali A.R., Montalvao D. (2022). Effects of particle size on the separation efficiency in a rotary-drum eddy current separator. Powder Technol..

[15] Shan Z., Yuan Y., Cao B., Miao S., Li G., Wang Q. (2024). The effect of interaction between particles on eddy current separation. Sep. Purif. Technol..

[16] Fuller A., Fan Z., Day C., Barlow C. (2020). Digital twin: Enabling technologies, challenges and open research. IEEE Access.

[17] Botín-Sanabria D.M., Mihaita A.S., Peimbert-García R.E., Ramírez-Moreno M.A., Ramírez-Mendoza R.A., Lozoya-Santos J.d.J. (2022). Digital twin technology challenges and applications: A comprehensive review. Remote Sens..

[18] Yu W., Patros P., Young B., Klinac E., Walmsley T.G. (2022). Energy digital twin technology for industrial energy management: Classification, challenges and future. Renew. Sustain. Energy Rev..

[19] Zhang D., Gao X. (2022). A digital twin dosing system for iron reverse flotation. J. Manuf. Syst..

[20] Kender R., Rößler F., Wunderlich B., Pottmann M., Golubev D., Rehfeldt S., Klein H. (2022). Development of control strategies for an air separation unit with a divided wall column using a pressure-driven digital twin. Chem. Eng. Process.-Process Intensif..

[21] Guiqiang W., Junbao C., Chengzhang L., Shuo L. (2025). Edge-YOLO: Lightweight Multi-Scale Feature Extraction for Industrial Surface Inspection. IEEE Access.

[22] Hao X.l., Liang H. (2019). A multi-class support vector machine real-time detection system for surface damage of conveyor belts based on visual saliency. Measurement.

[23] Rahman M.A., Bakker M. (2013). Sensor-based control in eddy current separation of incinerator bottom ash. Waste Manag..

[24] Brooks L., Gaustad G., Gesing A., Mortvedt T., Freire F. (2019). Ferrous and non-ferrous recycling: Challenges and potential technology solutions. Waste Manag..

[25] Chamorro J., Vallejo L., Maynard C., Guevara S., Solorio J.A., Soto N., Singh K.V., Bhate U., Ravi Kumar G.V.V., García J. (2022). Health monitoring of a conveyor belt system using machine vision and real-time sensor data. CIRP J. Manuf. Sci. Technol..

[26] Ramly R., Sajak A.A.B., Rashid M. (2019). IoT recycle management system to support green city initiatives. Indones. J. Electr. Eng. Comput. Sci..

[27] Tabaghchi Milan S., Darbandi M., Jafari Navimipour N., Yalcın S. (2023). An energy-aware load balancing method for IoT-based smart recycling machines using an artificial chemical reaction optimization algorithm. Algorithms.

[28] Kia S., Leiding B. (2025). PiVisionSort: Integrating Image Processing and Machine Learning for Material Recognition on Conveyor Belts. Data, Information and Computing Science.

[29] Waheed F., Omar M., Ibrahim S.Z., Chugtai R., Aejaz S.H. (2023). Application of industrial IoT in developing a sustainable and automatic liquid filling plant. Pak. J. Sci. Res. (PJOSR).

[30] Bargal N., Deshpande A., Kulkarni R., Moghe R. (2016). PLC based object sorting automation. Int. Res. J. Eng. Technol. (IRJET).

[31] Almtireen N., Reddy V., Sutton M., Nedvidek A., Karn C., Ryalat M., Elmoaqet H., Rawashdeh N. (2025). PLC-Controlled Intelligent Conveyor System with AI-Enhanced Vision for Efficient Waste Sorting. Appl. Sci..

[32] Mustafa M.N. (2021). Classification of maintenance techniques and diagnosing failures methods. Proceedings of the Journal of Physics: Conference Series.

[33] Schröder M., Falk B., Schmitt R. (2016). Failure classification and analysis for technical products. Procedia CIRP.

[34] Lee W.Y. (2004). PLC Control of Scrap Battery and Lead Reclamation Process. Bachelor Thesis.

[35] Kasiemkhan A. (2013). Sensor Based Optimisation of Eddy Current Separation in Bottom Ash Recycling. Master’s Thesis.

[36] Aquib K., Fansupkar Y., Nath S., Gunde O. (2024). Automated Precision: PLC-Based Object Sorting System. Maharashtra State Board of Technical Education Sponsored Technical Paper Presentation Competition 2023–2024, Electronics and Telecommunication Engg. Group, Mumbai Region.

[37] Moray R., Pabalkar V. (2020). Creation of Smart Cities—Perception and Strategies towards Liveable Futures. Int. J. Innov. Creat. Change.

[38] Nagel J.R. (2018). An analytic model for eddy current separation. Miner. Eng..

[39] Zhang S., Forssberg E., Arvidson B., Moss W. (1999). Separation mechanisms and criteria of a rotating eddy-current separator operation. Resour. Conserv. Recycl..

[40] Yang H., Kuang Z., Zhu J., Xu F., Nie F., Sun S. (2022). Digital twin key technology on rare earth process. Sci. Rep..

[41] Yang Y., Miao C., Li X., Mei X. (2014). On-line conveyor belts inspection based on machine vision. Optik.

[42] Xianguo L., Lifang S., Zixu M., Can Z., Hangqi J. (2018). Laser-based on-line machine vision detection for longitudinal rip of conveyor belt. Optik.

[43] Zghaibeh M. (2023). A Blockchain-Based, Smart Contract and IoT-Enabled Recycling System. J. Br. Blockchain Assoc..

[44] Li M., Chen F., Zhou W. (2024). Digital-twin-based system for foam cleaning robots in spent fuel pools. Appl. Sci..

[45] Ruiz C., Dashti A. (2025). Mixed Models for Product Design Selection Based on Accelerated Degradation Testing. Proceedings of the 2025 Annual Reliability and Maintainability Symposium (RAMS).

[46] Li L., Aslam S., Wileman A., Perinpanayagam S. (2021). Digital twin in aerospace industry: A gentle introduction. IEEE Access.

[47] Kritzinger W., Karner M., Traar G., Henjes J., Sihn W. (2018). Digital Twin in manufacturing: A categorical literature review and classification. Ifac-PapersOnline.

[48] Sujatanagarjuna A., Kia S., Briechle D.F., Leiding B. (2023). MushR: A smart, automated, and scalable indoor harvesting system for gourmet mushrooms. Agriculture.

[49] Piromalis D., Kantaros A. (2022). Digital twins in the automotive industry: The road toward physical-digital convergence. Appl. Syst. Innov..

[50] Vachálek J., Bartalskỳ L., Rovnỳ O., Šišmišová D., Morháč M., Lokšík M. (2017). The digital twin of an industrial production line within the industry 4.0 concept. Proceedings of the 2017 21st International Conference on Process Control (PC).

[51] Chandan G., Jain A., Jain H., Mohana (2018). Real time object detection and tracking using Deep Learning and OpenCV. Proceedings of the 2018 International Conference on Inventive Research in Computing Applications (ICIRCA).

[52] Bradski G., Kaehler A. (2008). Learning OpenCV: Computer Vision with the OpenCV Library.

[53] Boyko N., Basystiuk O., Shakhovska N. (2018). Performance evaluation and comparison of software for face recognition, based on dlib and opencv library. Proceedings of the 2018 IEEE Second International Conference on Data Stream Mining & Processing (DSMP).

[54] Sharma A., Pathak J., Prakash M., Singh J. (2021). Object detection using OpenCV and python. Proceedings of the 2021 3rd International Conference on Advances in Computing, Communication Control and Networking (ICAC3N).

[55] Flusser J., Farokhi S., Höschl C., Suk T., Zitova B., Pedone M. (2015). Recognition of images degraded by Gaussian blur. IEEE Trans. Image Process..

[56] Tsomko E., Kim H., Izquierdo E. (2010). Linear Gaussian blur evolution for detection of blurry images. IET Image Process..

[57] Chityala R.N., Hoffmann K.R., Bednarek D.R., Rudin S. (2004). Region of interest (ROI) computed tomography. Proceedings of the Medical Imaging 2004: Physics of Medical Imaging.

[58] Khan I.H., Javaid M. (2022). Role of Internet of Things (IoT) in adoption of Industry 4.0. J. Ind. Integr. Manag..

[59] Rath K.C., Khang A., Roy D. (2024). The role of Internet of Things (IoT) technology in Industry 4.0 economy. Advanced IoT Technologies and Applications in the Industry 4.0 Digital Economy.

[60] Lampropoulos G., Siakas K., Anastasiadis T. (2019). Internet of things in the context of industry 4.0: An overview. Int. J. Entrep. Knowl..

[61] García G.B., Suarez O.D., Aranda J.L.E., Tercero J.S., Gracia I.S., Enano N.V. (2015). Learning Image Processing with OpenCV.

[62] Culjak I., Abram D., Pribanic T., Dzapo H., Cifrek M. (2012). A brief introduction to OpenCV. Proceedings of the 2012 35th International Convention MIPRO.

[63] Howse J. (2013). OpenCV Computer Vision with Python.

[64] Modrzyk N. (2018). Building Telegram Bots: Develop Bots in 12 Programming Languages Using the Telegram Bot API.

[65] Idhom M., Fauzi A., Alit R., Wahanani H.E. (2018). Implementation system telegram bot for monitoring Linux server. Proceedings of the International Conference on Science and Technology (ICST 2018).

[66] Jorge V.L., Bendaoud I., Soulié F., Bordreuil C. (2025). Deep learning-based YOLO for semantic segmentation and classification of weld pool thermal images. Int. J. Adv. Manuf. Technol..

[67] Toapaxi C.C., Eduardo C. (2019). Assessment Performance and Emittance Measurements Tests of Basler Digital Camera vs. the Standard BTV System at CLEAR.

[68] Bodenstorfer E., Hasani Y., Fürtler J., Brodersen J., Mayer K.J. (2012). High-speed line-scan camera with multi-line CMOS color sensor. Proceedings of the 2012 IEEE Computer Society Conference on Computer Vision and Pattern Recognition Workshops.

[69] Ciaglia F., Zuppichini F.S., Guerrie P., McQuade M., Solawetz J. (2022). Roboflow 100: A rich, multi-domain object detection benchmark. arXiv.

[70] Liang S., Wu H., Zhen L., Hua Q., Garg S., Kaddoum G., Hassan M.M., Yu K. (2022). Edge YOLO: Real-time intelligent object detection system based on edge-cloud cooperation in autonomous vehicles. IEEE Trans. Intell. Transp. Syst..

[71] Han B.G., Lee J.G., Lim K.T., Choi D.H. (2020). Design of a scalable and fast YOLO for edge-computing devices. Sensors.

[72] Bhavana N., Kodabagi M.M., Kumar B.M., Ajay P., Muthukumaran N., Ahilan A. (2024). POT-YOLO: Real-Time Road Potholes Detection using Edge Segmentation based Yolo V8 Network. IEEE Sensors J..

[73] Aboyomi D.D., Daniel C. (2023). A comparative analysis of modern object detection algorithms: YOLO vs. SSD vs. faster R-CNN. ITEJ (Inf. Technol. Eng. J.).

[74] Olorunshola O., Jemitola P., Ademuwagun A. (2023). Comparative study of some deep learning object detection algorithms: R-CNN, fast R-CNN, faster R-CNN, SSD, and YOLO. Nile J. Eng. Appl. Sci.

[75] Redmon J., Farhadi A. (2018). Yolov3: An incremental improvement. arXiv.

[76] Zhao Z.Q., Zheng P., Xu S.t., Wu X. (2019). Object detection with deep learning: A review. IEEE Trans. Neural Networks Learn. Syst..

[77] Wang C.Y., Bochkovskiy A., Liao H.Y.M. YOLOv7: Trainable bag-of-freebies sets new state-of-the-art for real-time object detectors. Proceedings of the IEEE/CVF Conference on Computer Vision and Pattern Recognition.

[78] Vlachos A., Bargiota E., Krinidis S., Papadimitriou K., Manglis A., Fourkiotou A., Tzovaras D. (2024). iblueCulture: Data Streaming and Object Detection in a Real-Time Video Streaming Underwater System. Remote Sens..

[79] Gomathi N., Sridevi I. (2015). Recovery of noble metal from E-waste using leaching, electro deposition and electro generative process. Der Pharma Chem..

[80] Peng T., Sellami S., Boucelma O., Chbeir R. (2023). Multi-output regression for imbalanced data stream. Expert Syst..

[81] Garreta R., Moncecchi G. (2013). Learning Scikit-Learn: Machine Learning in Python.

[82] Rodriguez-Galiano V., Sanchez-Castillo M., Chica-Olmo M., Chica-Rivas M. (2015). Machine learning predictive models for mineral prospectivity: An evaluation of neural networks, random forest, regression trees and support vector machines. Ore Geol. Rev..

